# Novel Insights into the Human Hsc70 Structure by Cross-Linking Mass Spectrometry and Molecular Modeling

**DOI:** 10.1021/acs.jproteome.6c00037

**Published:** 2026-07-29

**Authors:** Aleksandr Melikov, Vsevolod Viliuga, Daniel Kavan, Zdeněk Kukačka, Matthias P. Mayer, Petr Novák

**Affiliations:** † 86863 Institute of Microbiology, the Czech Academy of Sciences, Prague 14220, Czech Republic; ‡ Department of Biochemistry, Faculty of Science, Charles University, Prague 12820, Czech Republic; § Department of Biochemistry and Biophysics, Stockholm University, Stockholm 10405, Sweden; ∥ Science for Life Laboratory (SciLifeLab), Stockholm 17165, Sweden; ⊥ Max Planck Institute for Polymer Research, Mainz 55128, Germany; # Center for Molecular Biology of Heidelberg University (ZMBH), DKFZ-ZMBH-Alliance, Heidelberg 69120, Germany

**Keywords:** protein folding, homo-oligomerization, chaperone, Hsc70, mixed isotope cross-linking, structural mass spectrometry

## Abstract

Heat shock cognate protein 70 (Hsc70) is a 71 kDa molecular chaperone belonging to the Hsp70 family of heat shock proteins. These proteins act as ATP-dependent molecular machines that assist protein folding under both physiological and stress conditions such as hypoxia, heat shock, and pH fluctuations. In addition to general chaperone functions, Hsc70 performs specialized roles, including uncoating clathrin-coated vesicles, facilitating protein transport into organelles, and targeting proteins for lysosomal degradation. Members of the Hsp70 family are known to form dimers and higher oligomers, but the structural organization and functional relevance of these assemblies remain poorly understood. Earlier studies also suggested that J-domain proteins (JDPs) can promote Hsp70 dimerization. In this study, we used chemical cross-linking, high-resolution Fourier transform mass spectrometry (FTMS), ^15^N isotopic labeling, and advanced data analysis to investigate the structural organization of Hsc70 dimers. Cross-link-derived distance restraints enabled structural modeling of Hsc70 monomers and dimers using AlphaLink2. Our results reveal distinct ATP- and ADP-state dimer conformations that coexist in equilibrium. In the presence of the cochaperone DnaJB1, we observed a shift in the dimer–monomer equilibrium, accompanied by enhanced ATP hydrolysis and formation of intermediate species. These findings demonstrate that the Hsc70 dimer population is structurally heterogeneous and depends on nucleotide state and cochaperone interactions.

## Graphical abstract

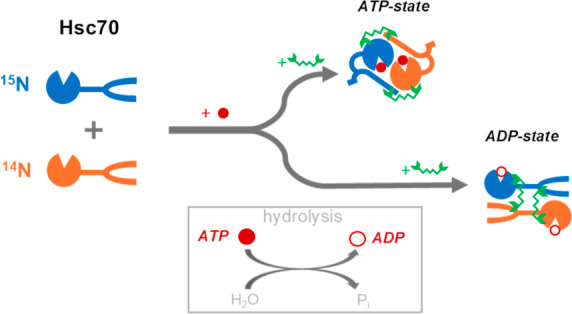

## Introduction

Heat shock protein 70 (Hsp70) is a ubiquitous protein family of ATP-driven molecular chaperones, which are indispensable for supporting the protein homeostasis in eukaryotic cells. Their main activity is to assist protein folding by binding and releasing the hydrophobic patches exposed in non-native protein conformations (e.g., present in partially folded proteins), providing the protein clients with more time to correctly fold and preventing them from aggregation.^1^ Every Hsp70 homologue shares a similar domain organization, which illustrates the high level of conservation of this protein family among all life domains. It is important to note that the term “Hsp70” is used to designate a protein family, unless stated otherwise. The N-terminal nucleotide-binding domain (NBD) is responsible for ATP binding and hydrolysis, providing the driving force for the Hsp70 cycle; this domain is connected to the substrate-binding domain (SBD), which has an affinity to the hydrophobic regions of client proteins and alternates between “open” and “closed” conformational states, therefore trapping and releasing a protein client.^1,2^ Two domains are structurally and allosterically connected by the short linker containing 4 hydrophobic residues in the middle part (e.g., Leu, Ile or Val), which is flanked by one Asp residue on each side.^3^ The C-terminal disordered tail of Hsp70 is believed to be responsible for interactions with various protein partners via the conserved EEVD motif (e.g., with Hop and Chip proteins).^4^ The functional cycle of each Hsp70 includes (1) the binding of a client by the SBD in the open state (i.e., ATP-bound); (2) ATP hydrolysis, SBD closure and trapping of the client; and (3) ATP/ADP exchange, opening the SBD and release of the client, which then may fold into the native state.^1,2,4^ Hsp70 proteins participate in many aspects of cell physiology, including protein homeostasis, transport into organelles, and targeting of misfolded proteins for degradation.^5–7^

Among them, Heat shock cognate protein 70 (Hsc70/HSPA8) is one of the 13 Hsp70 homologues encoded in the human genome and belongs to the first group of housekeeping chaperones.^2^ Hsc70 is a cytosolic protein that plays a significant role during the initial steps of protein folding. Particularly, the investigation of *de novo* folding during and immediately after translation revealed the yeast Hsp70 homologue, Ssa, being involved in the function of ribosome-associated chaperone machinery. The authors show that human Mpp11 can substitute for Zuo1 in the yeast RAC (ribosome-associated complex) by recruiting the multifunctional cytosolic yeast chaperone Ssa1 for the folding of nascent polypeptide chains as they exit the ribosomal tunnel.^8^ This finding led the authors to speculate that Hsc70 may fulfill the similar function in the cytosol of human cells, considering that no other plausible homologues are encoded in the human genome. In addition, Hsc70 participates in many other protein folding processes in human cells. For example, it serves as an uncoating enzyme for clathrin-coated vesicles; its interaction with Hsp90 is crucial for polypeptide transfer from the cytosol into the mitochondrial matrix and for maturation of a subset of clients including steroid hormone receptors; Hsc70 is required for β-importin transport into the cytosol, which is critical for the cytosol-nuclear transport cycle; and Hsc70 ensures the accumulation of cyclin D1 and assists the assembly of the cyclin D1/CDK4 complex, one of the cell cycle regulators.^9–13^ It is generally assumed that Hsc70 is an indispensable part of the cellular proteome.

Even though it is presumed that all Hsp70s act as monomers, several decades ago, published data suggested the existence of a dimeric form. During a gel filtration assay, Schmid et al.^14^ observed fractions that correspond to the Hsp70 dimer; moreover, upon the addition of ATP, the equilibrium drastically shifted from the dimer to the monomer, indicating the negative effect of ATP on the Hsp70 dimer stability. Nevertheless, the study of interactions of Hsp70 with its J-domain protein cochaperone (JDP) revealed that the fraction of the Hsp70 dimer has increased in the presence of both ATP and cochaperone; this was confirmed for multiple Hsp70 and JDP homologues, both by gel filtration analysis and native MS measurements.^15,16^ Further research on another Hsp70, the ER-resident BiP/HSPA5, showed that the BiP dimer formation is induced by thapsigargin-mediated Ca^2+^ depletion that induced the unfolded protein response because BiP became inactive. Dimerization and oligomerization were also observed in the wake of an unfolded protein response when misfolded proteins in the ER had been taken care of by refolding or “ER associated degradation”. These findings led to the “inactivation through oligomerization” hypothesis, which attempts to explain the rapid desensitization to the chaperone after the unfolded protein response was induced in the ER.^17–19^ Since then, multiple models of the Hsp70 dimer were proposed based on the various structural and biochemical data, including X-ray crystallography, XL-MS, native MS, and gel filtration combined with the specific proteolysis assay.^16,19–21^

Using Hsc70 as a model Hsp70 protein, we attempted to deduce the structures of its putative homodimers utilizing mixed isotope cross-linking (MIX) which enables to identify intra- and intermolecular distance constraints emerging from the reaction with the chemical cross-linker.^22,23^ To distinguish between inter- and intramolecular cross-links by mass spectrometry, the recombinant protein was expressed in both native and heavy nitrogen labeled forms since the intermolecular cross-links are identified using mixed-isotope cross-linking (MIX), where unlabeled (^14^N) protein (L-light) is mixed with ^15^N-labeled protein (H-heavy) in a 1:1 ratio before the cross-linking reaction. For intermolecular cross-links, triplet/quadruplet ion signals are present in the spectra (LL, LH, HL, and HH), whereas the intramolecular cross-links yield only doublet signals (LL and HH).^24^ However, quite often the “intermediate” interprotomer XLs can be detected in MS, for which the intensities of LH/HL forms are substantially lower than for LL and HH. For these types of XL, the calculation of inter/intra ratio is utilized according to the equation: (I_LH_ + I_HL_)/(I_LL_ + I_HH_), where I is the intensity of a corresponding XL-peptide. This parameter allows to define “rather inter” (the ratio ≥0.5) and “rather intra” XL (the ratio <0.5)^24^. Such an approach was utilized to obtain distance constraints for mapping the dimerization interface and structural rearrangement of Hsc70 upon ATP binding. These constraints were subsequently used for building structural models of Hsc70 homodimers *in silico*.

## Methods

### Protein Expression, Purification, and Isotopic Labeling

The bacterial plasmid vectors encoding human Hsc70 WT and DnaJB1 WT with N-terminal SUMO-His_6_-tag (pCA528 vector) were provided by the laboratory of Matthias P. Mayer (ZMBH, Heidelberg, Germany). The expression and purification were carried out based on the protocol reported earlier.^25^ The vectors were introduced into *Escherichia coli* BL21 Rosetta gami 2 (DE3) competent cells (Novagen, Germany) by chemical transformation. The transformed cells were grown in the M9 glucose minimal medium supplemented with either ^14^NH_4_Cl or ^15^NH_4_Cl correspondingly.^26^ To ensure that the proteins were produced in ^14^N- and ^15^N-forms, the cell passaging was performed in the corresponding media (3–4 times). In the final culture, the protein expression was induced by 1 mM IPTG overnight at 25 °C (at O.D. = 0.9). The expression of the proteins was verified by SDS-PAGE, before and after the IPTG induction. This research did not involve humans or animal participants.

After the induction, cells were harvested by centrifugation (14000g, 20 min, at 4 °C). The obtained pellets were resuspended in Tris lysis buffer (50 mM *Tris*–HCl (pH = 7.9), 300 mM NaCl, 5 mM MgCl_2_, 10% (v/v) glycerol, and sterile dH_2_O) supplemented with protease inhibitors (10 μg/mL aprotinin, 10 μg/mL leupeptin, 8 μg/mL pepstatin, and 1 mM PMSF) and DNase I (0.2 U/ml). For softer cell lysis, the EmulsiFlex-C3 High Pressure Homogenizer (Avestin, Canada) was used. After homogenization, the suspension was centrifuged (14000g, 20 min, at 4 °C), and the supernatant was collected.

The chromatographic steps of purification were done using an FPLC system NGC Quest 10 (Bio-Rad, USA). The protein was isolated using Ni-IMAC with gradient elution (EconoFit Nuvia columns, Ni-Charged, Bio-Rad, USA). The SUMO-His_6_-tag cleavage was performed using His-tagged recombinant SUMO protease Ulp1 (Sigma-Aldrich, USA) in the dialysis setup for the simultaneous buffer exchange into HKMG150 buffer (25 mM HEPES/NaOH (pH = 7.5), 150 mM KCl, 5 mM MgCl_2_, 10% (v/v) glycerol, and sterile dH_2_O) supplemented with 1 mM TCEP. The removal of the cleaved tag and Ulp1 protease was carried out by the second Ni-IMAC procedure. The obtained protein solution was concentrated to 2 mg/mL by centrifugation (MWCO 30 kDa), and 2 mL was injected for the SEC procedure (ENrich SEC 650 10 × 300 column, 24 mL, Bio-Rad, USA). The eluted fractions were analyzed by SDS-PAGE. The chosen fractions corresponding to the monomer were merged (Figure S1), concentrated, and flash-frozen in the liquid nitrogen, with the following storage at −80 °C.

### Mass Photometry

All the MP experiments were done on the Refeyn Two^MP^ mass photometer positioned on the Accurion vibration-isolation bench (Refeyn, UK). The calibration of the instrument was performed on the BSA solution and IgG solution (10 nM). The Hsc70 samples (20 μM) were incubated at room temperature overnight in HKM150 buffer (25 mM HEPES/NaOH (pH = 7.5), 150 mM KCl, 5 mM MgCl_2_, and LC–MS dH_2_O). Prior to the experiment, each protein sample was diluted to 10–20 nM and immediately measured. The ATP-time-dependent experiments were carried out by incubating samples with 40 μM ATP and measuring them in 10 min intervals for 90 min.

### Cross-Linking and Sample Preparation for LC–Mass Spectrometry

For each sample, ^14^N-Hsc70 and ^15^N-Hsc70 were mixed in equimolar ratio (total Hsc70 concentration 20 μM). The absolute protein concentration was determined spectrophotometrically at 280 nm (ε_280_ = 33350 M^–1^.cm^–1^). To establish the precise ^14^N:^15^N equimolar ratio, a small amount of each sample was digested with 0.5 μg of a Trypsin/Lys-C Mix (Promega, USA) for 30 min at 37 °C. The digested Hsc70 mixture was analyzed by MALDI-TOF to verify the relative ^14^N:^15^N abundance, and the concentration of each sample was adjusted correspondingly. For the experiments with the cochaperone, nonlabeled DnaJB1 was added to the Hsc70 mixture in a 1:10 and 1:2 molar ratio (DnaJB1:Hsc70). Prior to each cross-linking reaction, samples were incubated overnight at room temperature in HKM150 buffer supplemented with 1 mM TCEP; for the experiments with DnaJB1, the cochaperone was added freshly prior to the cross-linking reaction. Each sample was prepared in triplicate. For cross-linking, DSS (Thermo Scientific, USA) was utilized in the molar ratio of 1:50 (protein:reagent) based on the number of Lysine residues in the Hsc70 WT sequence, i.e., approximately 1 Lys per 1 DSS. Each Hsc70 sample was incubated with the corresponding ATP amount, and DSS was added at a certain time point. The cross-linking reaction was performed for 60 min at room temperature. For the dilution experiments, the higher starting concentrations of Hsc70 (50 μM) were incubated overnight in the same conditions described above. The adjusted amounts of ATP were added to the samples; immediately before the cross-linking reaction, the protein samples were diluted 50 times, and the adjusted amount of DSS was added (keeping the same protein:cross-linker = 1:50 molar ratio). The cross-linking reaction was performed at room temperature for 30 min and then quenched with ethanolamine (equimolar DSS/ethanolamine ratio).

After cross-linking, the additional reduction and blocking of cysteine side chains were performed (10 μL of XL sample +12.8 μL of buffer containing 15 mM TCEP, 62.5 mM 2-chloroacetamide, 120 mM 4-ethylmorpholine, 8% (v/v) acetonitrile, pH = 8.4). The untreated part of XL samples was used for the subsequent SDS-PAGE analysis. These chemical treatments were done at 70 °C for 5 min. After the samples were cooled to 37 °C, 1 μg of Trypsin/Lys-C Mix (Promega, USA) was added. The digestion was carried out overnight at 37 °C. The reaction was quenched the following morning by TFA (final concentration 0.1% (v/v)).

### LC–Mass Spectrometry Measurements

The LC–MS analysis was done by the UHPLC system Agilent 1290 Infinity II (Agilent, USA) connected to the inlet of 15T solariX FT-ICR (Bruker Daltonics, USA). 5 μL of the sample was injected by the Agilent 1290 autosampler onto the desalting precolumn (Luna Omega Polar C18 5 μm, 100 Å, 0.3 × 30 mm, PhenomeneX, USA) and desalted at a flow rate 20 μL/min for 5 min. Then, the desalted peptides were eluted into the analytical column (Luna Omega Polar C18 3 μm, 100 Å, 0.3 × 150 mm; PhenomeneX, USA) and separated at the flow rate of 10 μL/min at 50 °C in water–acetonitrile gradient (5–35%) for 35 min (maximal pressure 600 bar). Consequently, the column was washed with 95% acetonitrile for 5 min and equilibrated with 5% acetonitrile for 15 min. Mass spectra were acquired in positive mode over the *m*/*z* range 250–2500 with 10^6^ data points transient and 0.2 s ion accumulation with two scans averaged per spectrum. The MS measurements were performed in the FT-ICR operating software, ftmsControl 2.1.0.

### Data Analysis of Identified XL-Peptides

The acquired MS-spectra were interpreted with Data Analysis 4.4 (Bruker Daltonics, USA) and LinX 2.0 softwares.^24^ Briefly, the LC–MS data were deconvoluted by SNAP 2.0 algorithm and exported in Data Analysis into.txt format, which served as an input for LinX, where the cross-links identification is based on the high mass accuracy and number of nitrogens for each peptide. For search, LinX considered K, R specificity for trypsin, 4 mis-cleavages, fixed carbamidomethylation of cysteine, variable oxidation of methionine, and Type 0, 1, and 2 products for K-, S-, T-, N-terminus. Mass accuracy was kept below 1.5 ppm. The identifications were then exported to an Excel table, where the inter/intra ratio was calculated for every replicate. The ratios among replicates were then averaged, and the standard deviation was calculated. Then, the Venn diagrams were generated to filter out the cross-links found in all 3 replicates. In addition, we utilized a method for defining and calculating the XL relative occurrence according to the formula:

$$XL\;relative\;occurence=\frac{I\left(XL\right)}{I\left(standard\right)+I\left(XL\right)}$$

where I­(XL)—the base peak intensity of a XL peptide and I­(standard)—the base peak intensity of the reference peptide. An unmodified peptide, whose formation during tryptic digestion does not depend on the cross-linking reaction (originated from cleavage behind Arginine), served as a reference peptide. Both XL and reference intensity in this equation are only considered for LL or ^14^N–^14^N form for calculation purposes. Each replicate data was treated separately, the values were then averaged, and the standard deviation was calculated.

### Computational Modeling of Hsc70 Using Cross-Linking Distance Constraints

We modeled conformational states of monomeric and dimeric Hsc70 given the experimental constraints obtained from the cross-linking experiments using AlphaLink2.^27^ AlphaLink2 is a fine-tuned version of the AlphaFold-Multimer model that incorporates cross-linking distance constraints directly in the internal representation of the structural information on a given sequence input.^28^

We used distance constraints derived from cross-linking experiments under three experimental conditions. For modeling, the Hsc70 WT sequence was truncated from the C-terminal end (last 27 residues removed, therefore, the truncated sequence for modeling is 1-619 of Hsc70 WT). This was implemented due to the absence of C-terminal XL peptides in any spectra and the disordered nature of the C-terminal tail of Hsp70 homologues, which significantly increases the computing time for the prediction and produces low-confidence regions in a model. First, we modeled ATP- and ADP-induced monomeric Hsc70 states using constraints obtained from monomeric Hsc70 experiments; only intrachain distances were provided. Subsequently, we modeled the ATP- and ADP-induced dimeric Hsc70 states using intra- and interchain constraints obtained from the corresponding dimer experiments. Structure prediction was performed with a locally installed AlphaLink2 version (git commit 679d007) on a single NVIDIA A100 (40 GB) GPU. Multiple sequence alignments (MSAs) were generated with JackHMMER (via the AlphaLink2 helper script) against UniRef90/UniRef,^30^ MGnify, and bfd databases. For each cross-linking condition, we sampled 200 complexes with one recycling iteration. The dropout_crosslinks hyperparameter was set to 1.

When modeling dimers with ADP-derived cross-links under default, no MSA subsampling (*n*_eff_ = −1) settings, predictions, frequently converged to ATP-like conformations, consistent with ATP-derived cross-links rather than the intended ADP-state. To increase conformational diversity and remove the bias of AlphaLink2 toward ATP-state-induced predictions, we subsampled MSAs with *n*_eff_ = 100 and more aggressively with *n*_eff_ = 10. Aggressive subsampling yielded ADP-dimer models that better satisfied the target set of ADP-derived cross-link distance constraints, and we thus report ADP-induced dimeric states in the text produced with *n*_eff_ = 10 unless stated otherwise.

We computed Cα–Cα distances from the predicted structures for the residues that experimentally formed a cross-link and considered any cross-link constraint satisfied if the distance was below 35 Å. In the main text, we highlight the models with the lowest mean Cα–Cα distance across all cross-links. All predictions, MSAs, and analysis scripts are provided at https://zenodo.org/records/17945108.

## Results and Discussion

### Time-Dependent Mass Photometry

Since it is necessary to form ^14^N/^15^N Hsc70 heterodimers prior to the cross-linking experiments, mass photometry (MP) measurements were performed to assess the Hsc70 monomer–dimer equilibrium and its ATP dependency in solution. The acquired data allowed us to establish the timing for the following cross-linking (XL) reaction. From the collected data, the Hsc70 dimer/monomer ratio was calculated and plotted against time (Figure S2). We observed a decrease in the Hsc70 dimer level during the first 20 min of the experiment, which can be attributed to ATP binding and conformational rearrangements of Hsc70, leading to dimer dissociation. However, as the intrinsic ATPase activity of Hsc70 allowed us to slowly convert ATP to ADP, the relative signal of the dimer started to gradually increase, reaching the maximum after 90 min. Therefore, we initially decided to choose 90 min as a time point for the XL reaction, where the majority of Hsc70 population supposedly adopts the^14,15^ N ADP-state (i.e., ADP-state condition). Nonetheless, this experimental design ensures the reduction of the preexisting homotypic ^14^N/^14^N and ^15^N/^15^N Hsc70 dimer population present in solution after the respective purification of the proteins and the formation of a newly assembled, more uniform heterotypic isotopically coded Hsc70 dimer population. To capture the different conformational states of Hsc70 and potentially transient ATP dimeric states, we decided to perform XL reactions at 10 min as well, where the majority of Hsc70 should be present in the ATP-bound state. To ensure the abundance of the ATP-state, an excess of ATP (Hsc70:ATP = 1:50) was used for this experiment.

### Intramolecular Cross-Links and Hsc70 Monomer Modeling

The analysis of 100% intramolecular cross-links (i.e., without LH/HL peptide forms detected) revealed 72 sites for the ATP-state and 64 for the ADP-state conditions, which were identified in all replicates (Figure S3A,B). Among them, 34 XLs were common between the two conditions (Figure 1A, **left**), representing almost 33% of the total XL number. In addition, a substantial fraction (∼25% of total) of XL sites is located within ≤20 amino acids on the Hsc70 primary sequence (Table S1), indicating the possibility of proximity effect and depletion of the XL reagent on near neighboring lysine residues. To increase the amount of ADP- and ATP-unique interprotomer XLs and suppress the uninformative interdimer XLs, dilution XL experiments were performed as well. Indeed, the overall number of XLs found was reduced from 102 to 60 (Figures S3C,D and 1A, **right**), with short-distanced XL fraction reduced to 11.7% (Table S1). On the contrary, the fraction of common, nonunique XLs has increased (34 of 60 total), even more complicating their assignment to one state or another. Even though clear differences are seen between the two experimental conditions and both states of Hsc70 can be distinguished, it is evident that Hsc70 ATP- and ADP-states coexist in an equilibrium in solution for a substantial amount of time. The prolonged ATP/ADP exchange by Hsc70 is underlined by very slow intrinsic ATPase activity of Hsp70 proteins: e.g., for the bacterial DnaK homologue, the determined rate constant for the intrinsic ATPase activity is 6 × 10^–4^ s^–1^ corresponding to *t*_1/2_ ≈ 20 min^21,29^ and for human Hsc70 2.38 × 10^–3^ s^–1^; *t*_1/2_ ≈ 4.7 min.^30^ The ATPase rate of Hsc70 is not significantly affected by the ADP dissociation rate, as it is for bacterial Hsp70 homologues (for bacterial DnaK and bovine Hsc70, *t*_1/2_ = 495 and 22.5 s, respectively).^31^ This supported the requirements of the XL semiquantification method, which would allow us to disentangle the detected XLs and assign it to different Hsc70 conformations.

**Figure 1:**
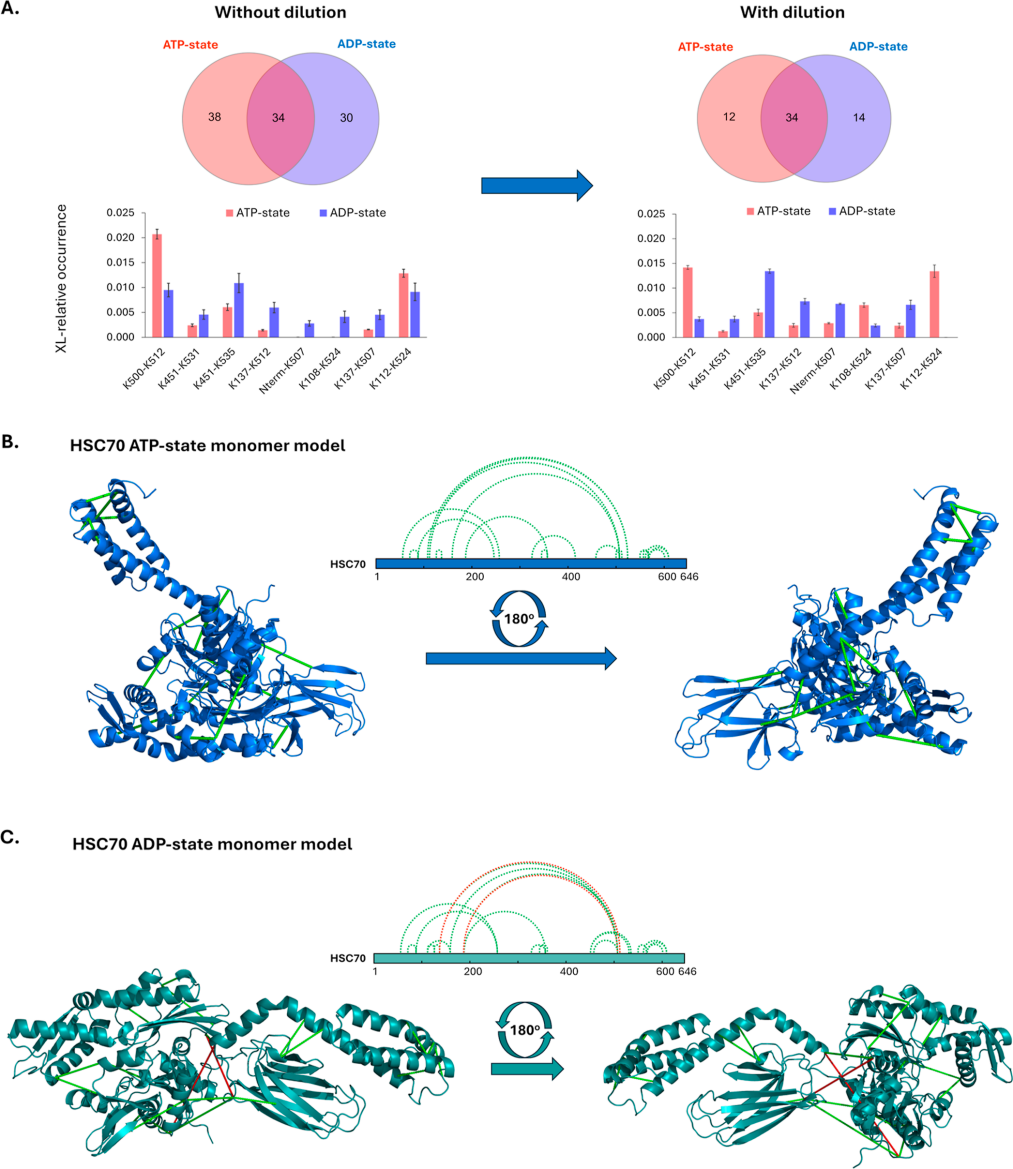
Analysis of identified Hsc70 intra-XLs and monomer conformation prediction results. (**A**) The comparison between intra-XLs sets identified in ATP-state and ADP-state conditions using a standard cross-linking pipeline, i.e., without dilution (**left**) and with dilution applied (**right**); the diluting pipeline allowed to reduce the total number of identified XLs and revealed certain changes in XL relative occurrences, which simplified the XL assignment to ATP- and ADP-conformation. (**B**,**C**) The best predicted model of Hsc70 with the corresponding XL map for in ATP-conformation (**B**) and ADP-conformation (**C**). The green color represents nonviolated distance constraints (35 Å cutoff), the red—violated.

For semiquantification of relative XL occurrence, an internal standard was used—the tryptic Hsc70 peptide T37-R49 (R̂TTPSYVAFTDTER̂L). Since the cross-linked Lys residues represent a steric hindrance for trypsin, which primarily cleaves after Lys and Arg, the crucial criterion for the standard peptide of choice was the Arg-only cleavage origin.^32^ Therefore, the enzymatic cleavage of such peptides is not affected by cross-linking, and their intensity in MS will vary solely with overall digestion efficacy, changing instrumental conditions or overall ionization efficiency. Normalization was then applied to every XL-peptide found across a given triplicate using the formula described in the Methods section. Comparing the values between ATP- and ADP-state experiments, notable differences in XL occurrences are seen, often with >1.5-fold change (Figure 1B). A dilution effect on relative quantification was observed as well, which, depending on a particular XL site, is comprised of (a) increase of difference in XL occurrence average values; (b) complete disappearance of the XL-peptide signal at one of the conditions; and (c) reverse in occurrence dominance (e.g., an XL more abundant in the ATP-state condition is suppressed in the same condition after dilution was applied). However, for a subset of XL-sites, little or no change (<1.2-fold change) in the abundances between ATP- and ADP-condition were determined, indicating their indifference for the protein conformational shifts. These criteria allowed us to distinguish the most significant XLs and assign them to ATP or ADP-state of Hsc70. The full list of identified intra-XLs is included in Table S1.

The processed XL distance constraints were provided as input to AlphaLink2 to model ATP- and ADP-induced monomeric states of Hsc70 (see Methods for more details). To avoid model overconstraining and biasing the structure toward incompatible constraints, the final XL sets for prediction were selected based on the XL occurrences and random sampling (Table S1). For modeling the Hsc70 ATP-state monomer, 18 XLs were selected; for modeling the ADP-state −21. AlphaLink2 predicted.

ATP-bound conformations that satisfied 18/18 experimental XLs and ADP-bound conformations that satisfied 19/21 (with K137–K512, 39.0 Å, and K187–K507, 37.0 Å) with high confidence (Figures 1B,C and S5). Predicted conformations are distinct from each other and are consistent with the structures of Hsp70 homologues published earlier. For instance, our ATP-state model (Figure 1B), the predicted Hsc70 conformation, is very similar to structures of *T. thermophila* ATP-Ssb1 (PDB code 5TKY, Cα RMSD = 1.52 Å^2^) and to the *E. coli* ATP-DnaK (PDB code 4JNE, Cα RMSD = 1.61 Å^2^), both derived from X-ray diffraction.^33,34^ ATP-DnaK, as well as ATP-Hsc70, is characterized by opened SBD that ensures the subsequent protein client binding, which induces ATP hydrolysis and the conformational shift into the ADP-state.^21^ This highlights the significant structural conservation across the different domains of life. For the ADP-state model, the key conformational shift resides in SBDα-lid docking onto SBDβ, creating a locked domain with a potential protein client trapped in it, which we have successfully predicted with the selected distance constraints (Figure 1C). However, the mutual flexibility of NBD and SBD in ADP-state creates a wide range of putative conformations (“2 balls on a string”) and complicates a precise modeling of this state; hence, the reported model is considered an “averaged” ADP-state arrangement, rather than a particular Hsc70 conformation. A crucial experimental structure, which serves as a reference to our model, is the ADP-conformation of *E. coli* DnaK, derived from NMR residual dipolar coupling and spin labeling data (PDB code 2KHO).^35^ Subsequently, NBD and SBD of this protein model were separately superimposed on the obtained model, showing very high degree of homology (Cα RMSD [NBD] = 1.19 Å^2^, Cα RMSD [SBD] = 0.75 Å^2^). The distance constraint distributions for the monomer models shown here are depicted in Figure S6. For the additional validation of the obtained models, the permuted XL distances were analyzed as well (i.e., XL distances used for ADP-state modeling were mapped on the ATP-model and vice versa), with 16/21 permuted XLs satisfied in the ATP-state model and 16/18 for the ADP-model (Figure S8). A similar XL-MS strategy was utilized for structural studies of sugar cane Hsp70, SsHsp70: the results agree with our conclusions on highly dynamic nature of Hsp70 conformational changes.^36^ However, as no isotopic labeling was utilized, it is impossible to unambiguously deduce whether a given XL originates from the SsHsp70 monomer or dimer interface. This significantly contributes to the uncertainty in the XL assignment and questions the validity of the modeled structures.

After obtaining very promising models of Hsc70 ATP- and ADP-state monomer conformations, we proceeded to elucidate the putative Hsc70 dimer structures formed in solution under different nucleotide conditions.

### Intermolecular Cross-Links and Hsc70 Dimer Modeling

The novel strategies for XL reactions and data treatment were then applied to Hsc70 interprotomer XLs identified in the same experiments, combined with the ^14^N- and ^15^N-isotope labeling approach. In addition, the inter/intra ratio was calculated to verify the dimeric origin of the XL-sites and categorize the intermediate ones into “rather inter” and “rather intra” XLs.

The analysis revealed 33 and 19 interprotomer XLs for ATP and ADP condition without dilution, respectively (Figure S3E,F; Table S1). The significant overlap (∼33% of total) in identified XLs between the two conditions could suggest an ATP/ADP dimer equilibrium in solution (Figure 2, **left top**). An alternative explanation could be the molecular crowding due to the high protein concentrations. This suggestion is supported by calculated inter/intra ratio values, showing a prevalence for intra XLs. Comparing these data to the dilution experiments, a much lower number of total XLs is observed, with an ∼18% overlap (Figure 2, **right top)**. A substantial decrease in the XL number is found to ATP experiment, where after dilution only 5 inter-XL sites of much higher inter/intra ratios were detected (Figures S4H and 2, **bottom**). This effect might be attributed to the dilution XL experiment pipeline, which allows to suppress the number of transient, nonspecific interactions between protein molecules and enables the detection of Hsc70 dimers. This was not observed for the ADP condition, where nearly the same number of XL of a similar inter/intra ratio distribution was identified, compared to nondiluted experiments (Figures S4G and 2, **bottom**). The prevalence of “rather intra”-XLs, even after the dilution was applied, suggesting a more transient nature of ADP-dimers. This contradicts the substrate-like way of dimerization deduced from X-ray crystallography years ago, where the SBD of the first subunit binds the hydrophobic linker of the second subunit, creating a lock and potentially a stable asymmetric dimer.^20^ On the other hand, an Hsp70 antiparallel ADP-dimer conformation proposed by Morgner et al.^16^ from the earlier XL-MS experiments is more favorable due to purely noncovalent interactions and absence of the SBD-linker lock.

**Figure 2:**
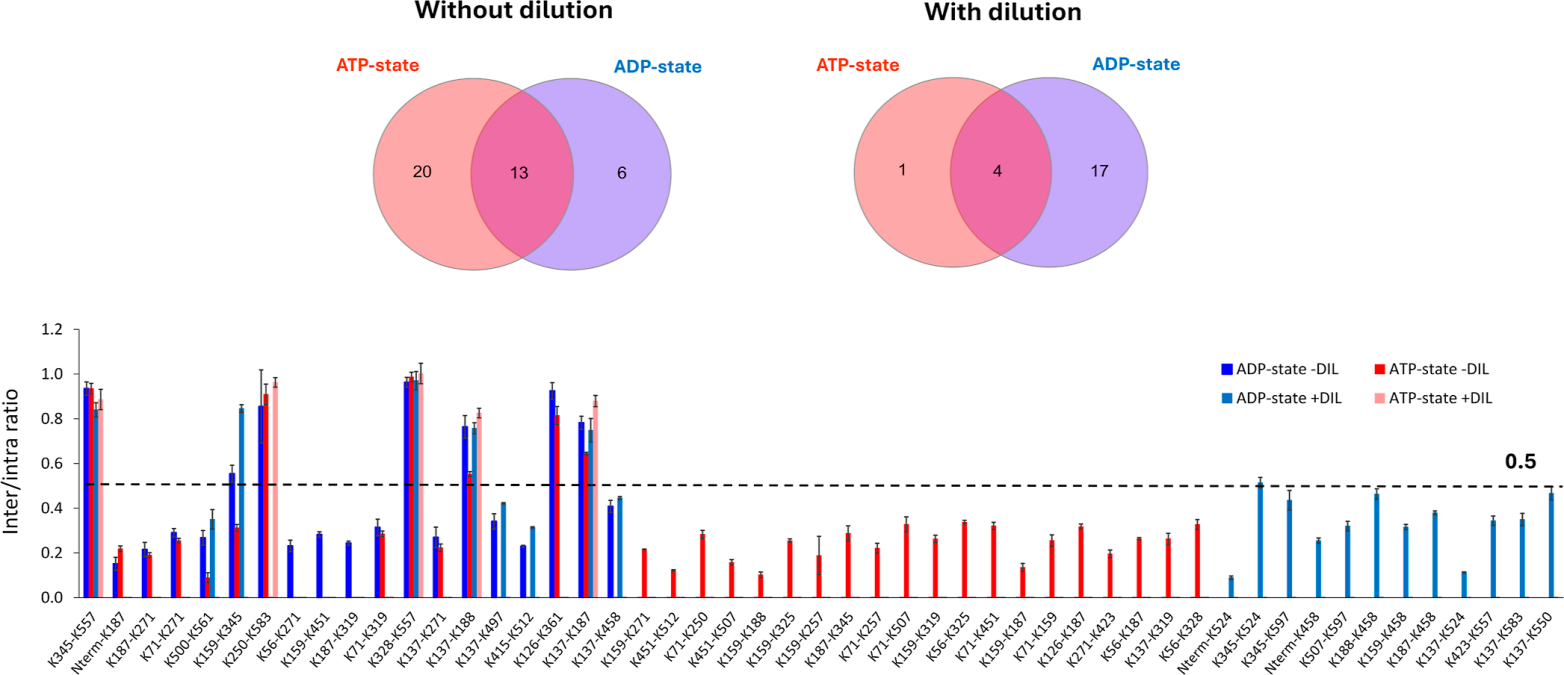
Analysis of intermolecular XLs from the same experiments. With the standard pipeline (without dilution, -DIL), a very high number of intermolecular XLs were identified in the ATP-state experiment, the majority of which fall below the 0.5 threshold of inter/intra ratio, suggesting a transient nature of these interactions. This was totally changed for ATP-state after the dilution (+DIL; 5 vs 33 XLs detected), where the remaining few XLs have relatively high inter/intra ratio value (all >0.5). For the ADP-state condition, the conclusion is not as straightforward: the dilution has only marginally changed the total number of XLs or the distribution of the ratio of inter/intramolecular XLs but led to identification of novel XLs.

For Hsc70 dimer modeling, both intra- and intermolecular XLs were utilized as distance constraints. For modeling the ATP-state dimer, the same set of intrachain XLs was utilized; as interchain constraints, the XLs identified in the ATP-state dilution experiment were used, excluding only one (K137–K188, as K137–K187 was identified as well and considered synonymous; Table S1). The interprotomer XL assignments to the ATP-state were also confirmed by the corresponding XL relative occurrences. The intra-XLs were applied on both chain A and B for retaining similar subunit configuration; the interprotomer XLs were applied in both polarities, therefore implying a symmetrical subunit interface. The produced high-confident output model did not violate any of the applied distance constraints (36/36 intra and 8/8 inter) and is consistent with the early identified Hsp70 ATP-dimer, creating the NBD_(A)_-NBD_(B)_ interface and additionally stabilized by SBDα_(A)_-NBD_(B)_/SBDα_(B)_-NBD_(A)_ contacts (Figures 3A and S5 and S6).^21^ Modeling of the ADP-state dimer with the similarly selected intra- and inter-XLs was more challenging, with modeling driven to the ATP-like conformation. Random sampling of XL-sites and reducing the distance constraint sets allowed us to obtain antiparallel ADP-dimer configuration (Figure 3B, **top**), with 14/16 intra- and 14/16 inter-XL constraints satisfied. The modeled antiparallel conformation agrees with the findings of Morgner et al., who utilized XL-MS on the human Hsp70 homologue.^16^ However, further subsampling of distance constraints and attempts for nonsymmetric ADP dimer modeling (e.g., substrate-like binding dimer) failed: even with a minimal set of constraints ensuring SBD closure (K451–K531 and K451–K535) and intersubunit contacts, the constraints are still majorly violated (Figure 3B, **bottom**). A selected minimal constraint set (Table S3) is however theoretically compatible with the substrate-like binding dimer proposed by Chang et al.^20^ (Figure 3B, **right**). The attempts to support this conformation by XL-driven structure modeling were not successful due to several possible reasons. Default AlphaLink2 predictions without aggressive MSA subsampling systematically relaxed ADP-dimer target conformations into ATP-like states, i.e., they satisfied ATP- rather than ADP-derived cross-links (Figure S6). This suggests that sequence homologues of Hsc70 impose a strong structural bias toward the ATP-bound state, rendering the ADP conformation of a dimer effectively out-of-distribution. Even though aggressive subsampling (*n*_eff_ = 10) produced ADP-like dimers that best matched the ADP cross-link constraints, the resulting models remained heterogeneous and did not consistently satisfy all constraints. The existence of nonsymmetric dimer and possibility of alternative dimerization modes are underlined by earlier studies on self-association of isolated Hsc70 SBD with interdomain linker attached (residues 385-646).^37^ As isolated NBD alone could not oligomerize even at elevated concentrations, the substrate-like dimerization mechanism involving recognition of the interdomain linker as a peptide substrate by SBD could not be eliminated. However, our findings are not compatible with SBDβ-alone oligomerization (due to the absence of multiple inter-SBDβ-XLs), which was observed by Fouchaq et al.^38^ In the data sets obtained, there is clear evidence of involvement of interactions between SBDs in parallel (Figure 3B) and potentially substrate-like dimerization mode of Hsc70. The problem with alternative dimer structure modeling may be potentially addressed by long-scale molecular dynamics, which would take into account the overall conformational changes of Hsc70 and the mutual flexibility of the domains in ADP-state. The identified XLs are fully compatible with multiple Hsc70 dimerization modes.

**Figure 3:**
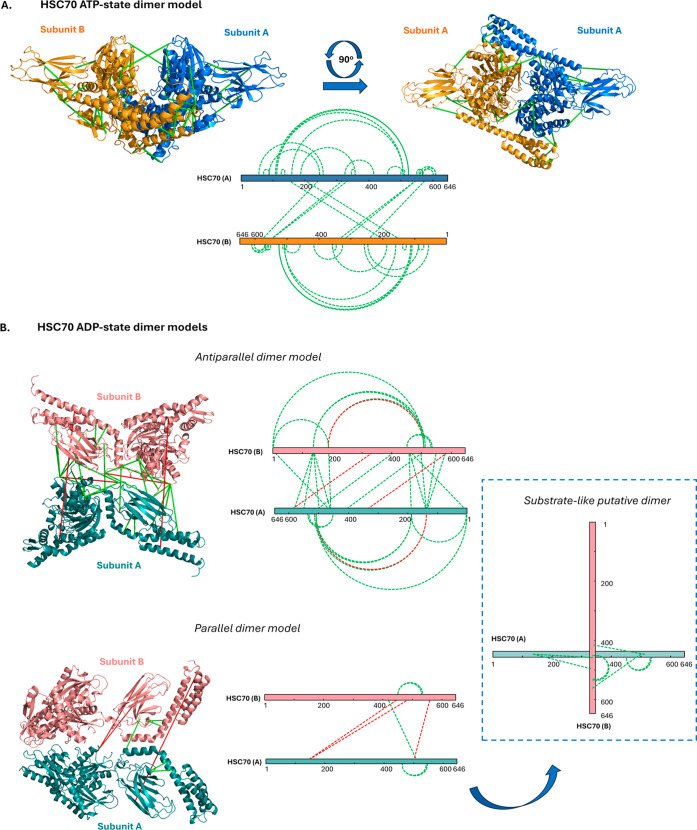
Predicted Hsc70 dimer models. (**A**) The ATP-state dimer model. Keeping the same set of intrachain constraints used for ATP-state monomer modeling and using detected interchain constraints, a dimer of the NBD–NBD interface was predicted with high confidence, with all constraints satisfied. (**B**) The ADP-state dimer models. Subsampling of XL-sets allowed us to model antiparallel dimer configuration (top); however, further reduction of distance constraint sets and attempts to model putative nonsymmetric dimer led to models with many constraints violated (bottom). The minimal XL set identified for ADP-state dimer are, however, theoretically compatible with the substrate-like binding dimer proposed by Chang et al. earlier.^19^

The proposed models of human Hsc70 dimers and our findings are coherent with the earlier studies conducted on other Hsp70 homologues: the formation of ATP dimer of similar topology was observed for bacterial DnaK by X-ray crystallography and additionally confirmed by native MS, gel filtration, and specific proteolysis essay.^34,39^ It was established that introduction of mutations compromising the dimer formation does not affect the ATP-allosteric coupling but significantly diminishes the chaperoning activity and the interactions with Hsp40 cochaperone, DnaJ.^39^ The authors emphasize the importance of the transient ATP DnaK dimer for the functionality of chaperone cycle. Similarly, stress-inducible Hsp70 human homologue is prone to association into dimer under ATP conditions, which is crucial for its interactions with Hsp40 and other protein cofactors (e.g., Chip, a E3 ubiquitin ligase).^40^ It is noteworthy that the authors reported that in the Apo form, neither DnaK nor human Hsp70 are oligomeric, and only a small fraction of human Hsc70 appears to be dimeric.^40^ These findings are at odds with previous discoveries in the field.^15,16,41,42^ In contrast, on addition of 200 μM ATP to 40 μM Hsp70, Trcka et al.^40^ observed almost 100% dimer fraction for Hsp70 and even a trimer for Hsc70, which contradicts the resolved crystal structures and our own observations. The difference in the dimer/monomer profile may originate from the protein construct used in the mentioned study and our own experiments: the Hsc70 proteins used in this study are untagged, with authentic N-termini, whereas the proteins used in Trcka et al.^40^ contained an N-terminal extension of 31 amino acids, as derived from their MS data (peptide list in the Supporting Information). This extension could affect the oligomerization behavior of Hsp70s in Apo and ATP-bound state observed by Trcka et al.^40^ Finally, our models of Hsc70 oligomerization created and distinguished by different ATP incubation conditions align well with the oligomerization network established years ago and expands it: ATP binding is responsible for the immediate conformational changes and consequent subunit dissociation; upon conversion of ATP to ADP by Hsc70 intrinsic ATPase activity, monomeric subunits tend to reassociate into oligomers.^43^ However, the role of peptide substrate binding was not tackled in this work; the current study could be potentially expanded by studying the effect of isotopically labeled peptide substrates.

The shared mode of association identified for various Hsp70 homologues highlights the high degree of evolutionary conservation and physiological meaning of Hsp70 dimer assemblies. It is additionally supported by investigation of role of PTMs on Hsp70 dimerization and studies of Hsp70 interactome dynamics under stressful conditions.^44,45^

### Effect of DnaJB1 Cochaperone on Hsc70 ATP/ADP-Equilibrium

Two different cochaperone concentrations (2 μM—low DnaJB1, and 10 μM, high DnaJB1; the numbers reflect total dimeric DnaJB1 concentration) were tested to verify the effect of DnaJB1 on Hsc70 ATP turnover and dimerization. For these experiments, higher amounts of ATP were used (Hsc70:ATP = 1:50) to prolong the existence of the ATP-state of Hsc70 in the context of DnaJB1-enhanced ATP hydrolysis. The proteins were then cross-linked after 10 min upon the ATP addition. The analysis of the obtained XL sets was divided into several subcategories, based on their origin: (a) Hsc70 intramolecular XLs; (b) Hsc70-Hsc70 interprotomer XLs; and (c) Hsc70-DnaJB1 intermolecular XLs. The first two groups were processed in a similar manner to Hsc70-only experiments above and compared to them (Tables S1 and S2). The cross-links between Hsc70 and DnaJB1 were selected based on the presence of both LL and HL XL-peptide signals in the spectra due to the presence of both ^14^N- and ^15^N-form of Hsc70 and only ^14^N-form of DnaJB1 (Table S2).

A total of 50 and 38 intramolecular XLs were identified for low- and high-DnaJB1, respectively, with 35 XLs in common between the two experiments (Figures S4A,F and 4A, **left**). The prominent overlap between the XLs (∼67% of total) is most likely due to the similar incubation and cross-linking conditions; it also confirms that even catalytical amounts of the cochaperone are sufficient to induce corresponding Hsc70 conformations. Interestingly, lower number of XLs was detected for the high-DnaJB1 experiment, probably indicating a higher cross-linker consumption by the elevated DnaJB1 concentration. When compared to ATP- and ADP-state experiments described earlier (without dilution), a higher overlap with the ADP-state experiment is seen as expected from the ATP-hydrolysis stimulating activity of DnaJB1 (Figure 4A, **right**). The relative quantification of the XLs (filtered by ANOVA test with FDR-corrected p-value) further supports the similarities between ADP-state and DnaJB1-containing conditions (Figure 4A, **bottom;** Tables S2 and S3): it is evident that higher concentrations of cochaperone favor XL sites, which are more characteristic of the ADP-state (e.g., K451–K535, K137–K512, and K137–507). However, in some cases, a XL is suppressed/enhanced even more, and its occurrence is rather different from earlier Hsc70-only experiments (e.g., K500–K512, N_term_-K507, and K112–K524); in other instances, the calculated occurrences fall in between the values of ATP- and ADP-state (e.g., K451–K531 and K108–K524).

**Figure 4:**
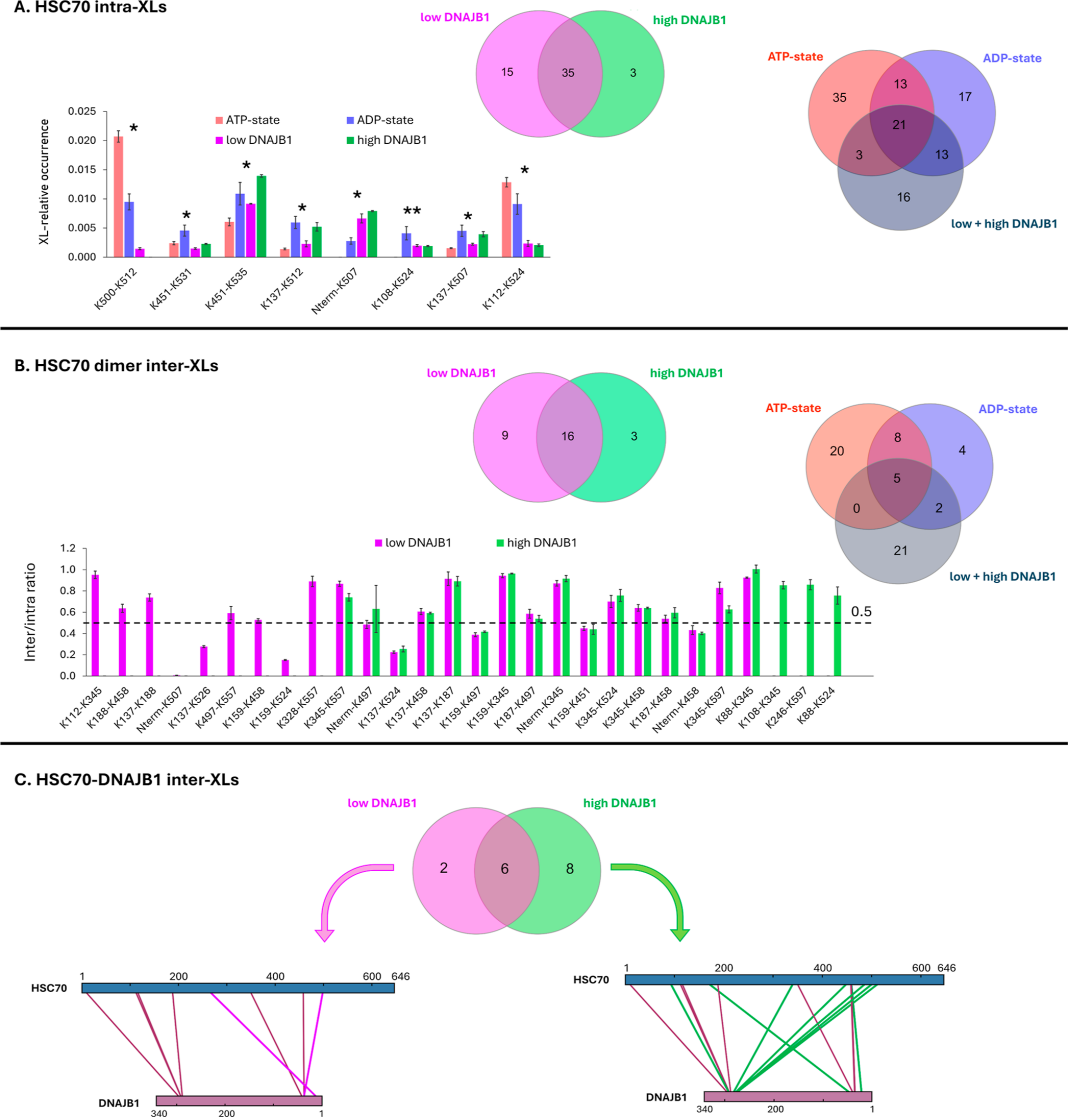
Analysis of XLs from the DnaJB1 experiments. (**A**) Hsc70 intra-XLs in the presence of DnaJB1 in different concentrations. DnaJB1 effectively induces or suppresses the formation of certain Hsc70 XLs earlier assigned to the ADP-state of Hsc70; however, this is not the case for all of them, suggesting the formation of Hsc70 conformations different from experiments in the absence of DnaJB1 (i.e., ATP-state and ADP-state). Asterisks indicate XLs with significant changes across conditions (one-way ANOVA, with FDR-corrected *p*-value; * *p* < 0.005, ** *p* < 0.05). (**B**) Hsc70 inter-XLs in the presence of DnaJB1. The majority of the detected Hsc70-Hsc70 XLs was not identified in the absence of DnaJB1; for the XLs shared between low and high DnaJB1 conditions, no significant shifts in inter/intra ratio values were observed. (**C**) Hsc70-DnaJB1 interprotein XLs. The XLs found in both conditions (dark red) and at low DnaJB1 (magenta) indicate J-domain binding to the Hsc70 interdomain junction and the NBD and CTD2 binding to the NBD of Hsc70. At elevated DnaJB1 concentrations, additional contacts of the CTD2 across the whole Hsc70 sequence (green) were observed, suggesting either nonspecific, transient interactions, or a digestion bias (almost all the additional XLs bear the same CTD2-derived DnaJB1 peptide).

Therefore, from our data, it is difficult to ultimately conclude that DnaJB1 alone can shift the equilibrium of Hsc70 toward the ADP-state in the absence of a protein substrate. The population in the solution seems to represent an array of intermediate Hsc70 states, transiently interacting with DnaJB1 and stimulating its ATPase activity.

Similarly, several intermolecular Hsc70 XLs were identified in the same data sets—25 at low DnaJB1 and 19 at high DnaJB1, with ∼57% overlap (Figures 4B, **left** and S4C,D). The analysis of inter/intra ratio values revealed no significant changes between the two conditions, indicating the similarities between Hsc70 dimer populations (Figure 4B, **bottom**). The relative quantification confirmed this hypothesis, with the values being very close to low and high concentrations of DnaJB1 (Table S2). Nevertheless, when compared to ATP- and ADP-state Hsc70 interprotomer XLs, no major overlap with either experiment is observed, with over 20 unique dimer XLs observed in the presence of DnaJB1 (Figure 4B, **right**). Within the little overlap with Hsc70-only experiments, intermolecular XLs affected by DnaJB1 exhibit no consistent trend: some XLs are more frequent compared to both ATP and ADP-state (e.g., K159–K345), some occur with similar intensity as in the ATP-state (e.g., K345–K557), some with similar intensity as in the ADP-state (e.g., K137–K458), and some are suppressed compared to the ATP- and ADP-state (e.g., K137–K187/188). Our data indicate different dimer structures forming in the presence of the cochaperone via interactions involving the J-domain and the existence of putative ternary complexes consisting of the Hsc70 dimer and the DnaJB1 native dimer.

The interaction between Hsc70 and DnaJB1 was then investigated by searching for corresponding Hsc70-DnaJB1 intermolecular XLs. In total, 6 and 14 XLs between the two proteins (37.5% overlap) were identified for low DnaJB1 and high DnaJB1 experiments, respectively (Figures 4C and S4E,F; Table S3). The N-terminal J-domain was mainly found to interact with the Hsc70 interdomain junction, consisting of the NBD interface and SBDβ interface (e.g., K345–K35, K458–K35, K500–K37, and K159–K35) These findings are in the agreement with the JDP binding mode to Hsp70 proteins, established and confirmed by the crystal structure of bacterial Hsp70, DnaK, with the J-domain of the bacterial JDP, DnaJ.^46^ The identified XLs at this interface were then mapped onto the created homology model of the Hsc70 + DnaJB1-JD complex using the crystal structure as a template (PDB 5NRO, Figure 5). The XL map and the distance information confirmed the position of JD at the Hsc70 interdomain junction, with a pair of XLs indicating the dynamics of JD interaction with the chaperone. In addition, multiple XL-peptides composed of a similar fragment of DnaJB1 (i.e., containing residue K286) were identified in the spectra as well. The contacts between DnaJB1 C-terminal binding domain 2 (CTD2) and various regions of Hsc70 do not exhibit a particular pattern: at low DnaJB1 concentration, the contacts between Hsc70 NBD and DnaJB1 CTD2 were mostly detected, whereas at higher DnaJB1 concentration, we observe some additional contacts of CTD2 with Hsc70 interdomain junction and SBD (Figure 4C). It is certainly possible that Hsc70 could be recognized as a protein substrate by DnaJB1 and therefore be bound by CTD2. However, due to high prevalence of XL-peptides containing the same DnaJB1 cross-linked residue (K286), the nonspecific nature of these interactions or tryptic digestion bias can be considered as well. The higher number of XLs was expected for the elevated amount of DnaJB1 added to the mixture, and the additional sites detected may correspond to nonspecific protein interactions due to molecular crowding.

**Figure 5:**
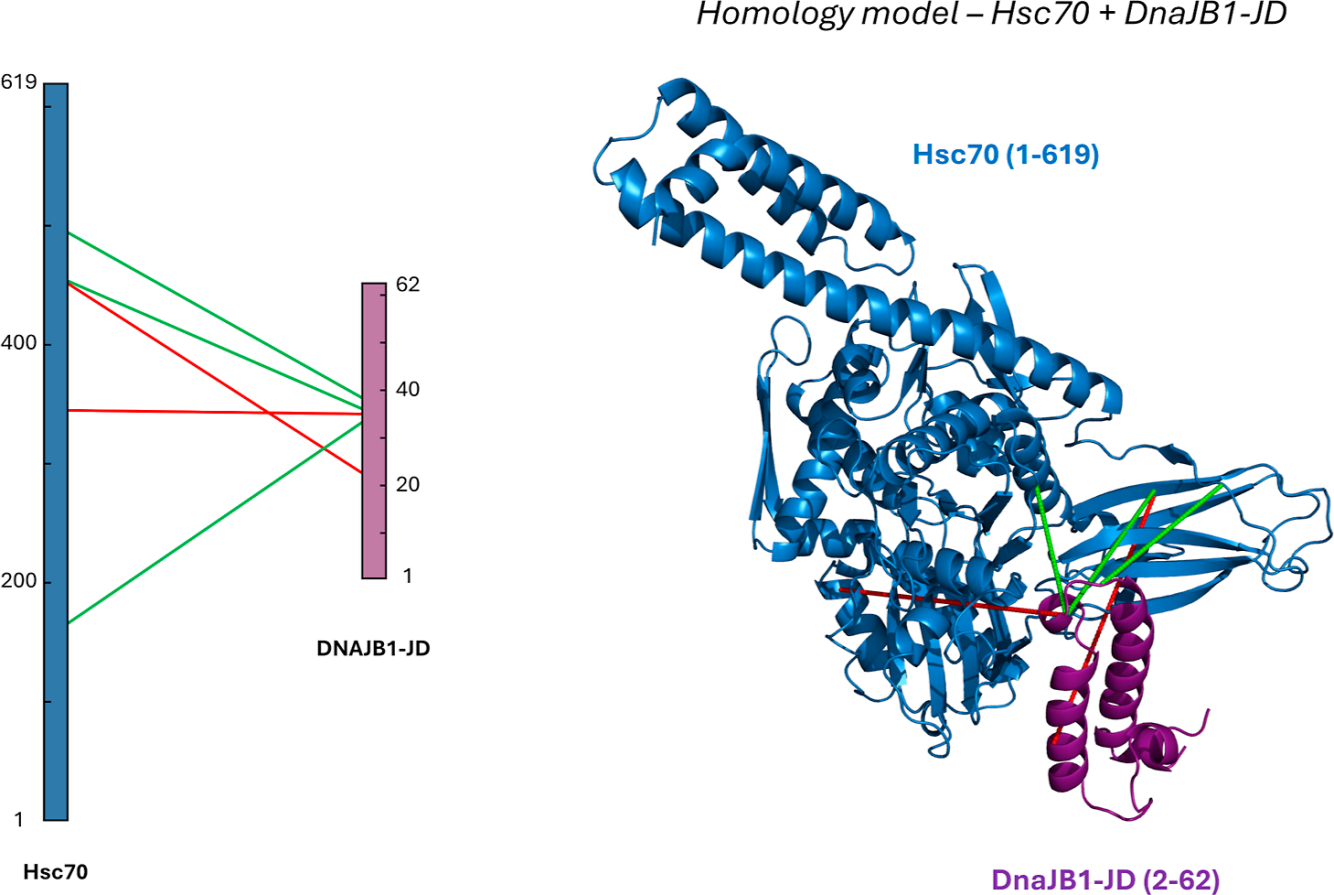
Homology modeling results of truncated Hsc70 (1-619) and DnaJB1-JD (2-62) with the corresponding interprotein XLs identified among low-DnaJB1 and high-DnaJB1 experiments. The modeling was done using the crystal structure of the DnaK + DnaJ-JD complex (PDB 5NRO), excluding the XL-distance constraints. In total, five XLs were identified at the interface between Hsc70 and N-terminal JD, with three satisfying the distance threshold (K458–K35, K500–K37, and K159–K35; in green) and two not satisfying (K345–K35, 43.7 Å, and K458–K21, and 40.2 Å; in red). The satisfied XLs locate JD precisely at the Hsc70 interdomain junction, which agrees with the crystal structure template; the remaining XLs may suggest the dynamics of Hsc70-JD interactions.

## Conclusion

The MIX strategy was employed to investigate the oligomerization and nucleotide-dependent conformational dynamics of human Hsc70 and to define the contribution of the cochaperone DnaJB1. A refined workflow combining rapid dilution prior to cross-linking with quantitative MIX analysis using an internal reference peptide substantially improved the resolution of XL-MS data, allowing reliable assignment of cross-links to the ATP- and ADP-bound states. This strategy is broadly applicable to studies of multiconformational homo-oligomeric protein complexes. Structure prediction tools capable of integrating XL-MS distance constraints into *ab initio* modeling pipelines (e.g., AlphaLink 2) provided valuable insights into Hsc70 structural dynamics. Incorporation of experimental constraints improved the model accuracy and biological relevance; however, flexible ADP-bound conformations remain difficult to capture. The broad conformational landscape of ADP-Hsc70 complicates XL-MS integration, precluding a single model that satisfies all of the cross-links.

Asymmetric homodimer configurations also remain challenging to predict due to biases in current modeling algorithms toward symmetric assemblies. Combining XL-MS constraints with molecular dynamics simulations may help address this limitation, although the large system size (>140 kDa) poses substantial computational demands.

Another important question resides in comparing our finding to in vivo conditions: although the solution conditions used were physiologically relevant (ionic strength, pH), the complexity of Hsc70 interactions in the cell was not reproduced in this work to the full extent. The previous findings on BiP oligomers confirm their presence in vivo.^19^ For Hsc70, it remains uncertain and strongly depends on the physiological state of the cell: for instance, in rapidly growing cells, it is conceivable that the abundance of potential chaperone clients may prevent the oligomerization; in the resting phase (G0), a surplus of Hsc70 may likely form oligomers, mediated by DNAJB1 or not. This topic remains to be investigated.

## Supplementary Material



## Data Availability

The mass spectrometry proteomics data have been deposited in the ProteomeXchange Consortium (http://proteomecentral.proteomexchange.org) via The PRIDE^47^ partner repository with the data set identifier PXD07223427.

## Notes

SEC chromatogram and SDS-PAGE gel of 14N-Hsc70 WT purification; Hsc70 WT dimer/monomer ratio upon addition of ATP in time, based on the MP experiments; Venn diagrams of replicate intercept for Hsc70-only XL-MS experiments; Venn diagrams of replicate intercept for Hsc70-DNAJB1 XL-MS experiments; prediction scores per residue (pLDDT) of the predicted Hsc70 models; distance restraint distributions of the predicted Hsc70 models; effect of MSA subsampling (n_eff) on the HSC70 dimer state; analysis of distances for permutated XL sets; total list of XLs identified in triplicates of the ATP-state and ADP-state experiments (with and without dilution) and their corresponding quantification (relative occurrence, inter/intra ratio); list of residues utilized for the Hsc70 monomer and dimer modeling; total list of XLs identified in triplicates of the ATP-state, ADP-state, low-DNAJB1, and high-DNAJB1 experiments (without dilution) and their corresponding quantification (relative occurrence, inter/intra ratio); ANOVA statistics for the Hsc70 intra-XL relative abundances from Table S2 (Hsc70 intra-XLs, Ambiguous XL); and modeling data and scripts can be found on https://zenodo.org/records/17945108 (PDF)

## ¶

Present Addresses Bio-Analytical Mass Spectrometry, University of Geneva, CMU, Geneva, Switzerland

## Abbreviations

ADP: adenosine diphosphate
ATP: adenosine triphosphate
DSS: disuccinimidyl suberate
Hsc70: human heat shock cognate protein 70
Hsp70: heat shock protein 70
JD: J-domain
JDP: J-domain protein
MIX: mixed isotope cross-linking
MP: mass photometry
MS: mass spectrometry
NBD: nucleotide-binding domain
N_term_: N-terminus of a protein
SBD: substrate-binding domain
XL: cross-linking.
