# Supplementary material for: Novel Insights into the Human Hsc70 Structure by Cross-Linking Mass Spectrometry and Molecular Modeling
Source: J Proteome Res. 2026 Jul 29;25(8):4005–17. doi: 10.1021/acs.jproteome.6c00037 (PMC13459546; doi:10.1021/acs.jproteome.6c00037)
Supplement: Supplementary file 1 [file pr6c00037_si_001.pdf]

NOVEL INSIGHTS INTO HUMAN Hsc70 STRUCTURE BY CROSS-LINKING MASS SPECTROMETRY AND MOLECULAR MODELLING.

ALEKSANDR MELIKOV[1,†], VSEVOLOD VILIUGA[3,4,5], DANIEL KAVAN[1], ZDENĚK KUKAČKA[1], MATTHIAS P. MAYER[6], PETR NOVÁK[1,2*]

[1] Institute of Microbiology, The Czech Academy of Sciences, 14220 Prague, Czech Republic;

[2] Department of Biochemistry, Faculty of Science, Charles University, 12820 Prague, Czech Republic;

[3] Department of Biochemistry and Biophysics, 10405 Stockholm University, Stockholm, Sweden;

[4] Science for Life Laboratory (SciLifeLab), 17165 Stockholm, Sweden;

[5] Max Planck Institute for Polymer Research, 55128 Mainz, Germany;

[6] Center for Molecular Biology of Heidelberg University (ZMBH), DKFZ-ZMBH-Alliance, 69120 Heidelberg, Germany

KEYWORDS: Protein folding, Homo-oligomerization, Chaperone, Hsc70, Mixed isotope cross-linking, Structural mass spectrometry.

*Correspondence to: pnovak@biomed.cas.cz

Supporting information

Table of contents

Additional Figures

1. **Figure S1:** The SEC chromatogram and SDS-PAGE gel of ^{14}N-Hsc70 WT purification.
2. **Figure S2:** The Hsc70 WT dimer/monomer ratio upon addition of ATP in time, based on the MP experiments.
3. **Figure S3:** Venn diagrams of replicate intercept for Hsc70-only XL-MS experiments.
4. **Figure S4:** Venn diagrams of replicate intercept for Hsc70-DNAJB1 XL-MS experiments.
5. **Figure S5:** Prediction scores per residue (pLDDT) of the predicted Hsc70 models.
6. **Figure S6:** Distance restraint distributions of the predicted Hsc70 models.
7. **Figure S7:** Effect of MSA subsampling (n_eff) on HSC70 dimer state.
8. **Figure S8:** Analysis of distances for permutated XL sets.

Tables

1. **Table S1:** Total list of XLs identified in triplicates of the ATP-state and ADP-state experiments (with and without dilution) and their corresponding quantification (relative occurrence, inter/intra ratio); the list of residues utilized for the Hsc70 monomer and dimer modelling.

2. **Table S2:** Total list of XLs identified in triplicates of the ATP-state, ADP-state, low-DNAJB1 and high-DNAJB1 experiments (without dilution) and their corresponding quantification (relative occurrence, inter/intra ratio).

3. **Table S3:** The ANOVA statistics for the Hsc70 intra-XL relative abundances from Table S2 (Hsc70 intra-XLs, Ambiguous XL).

A.

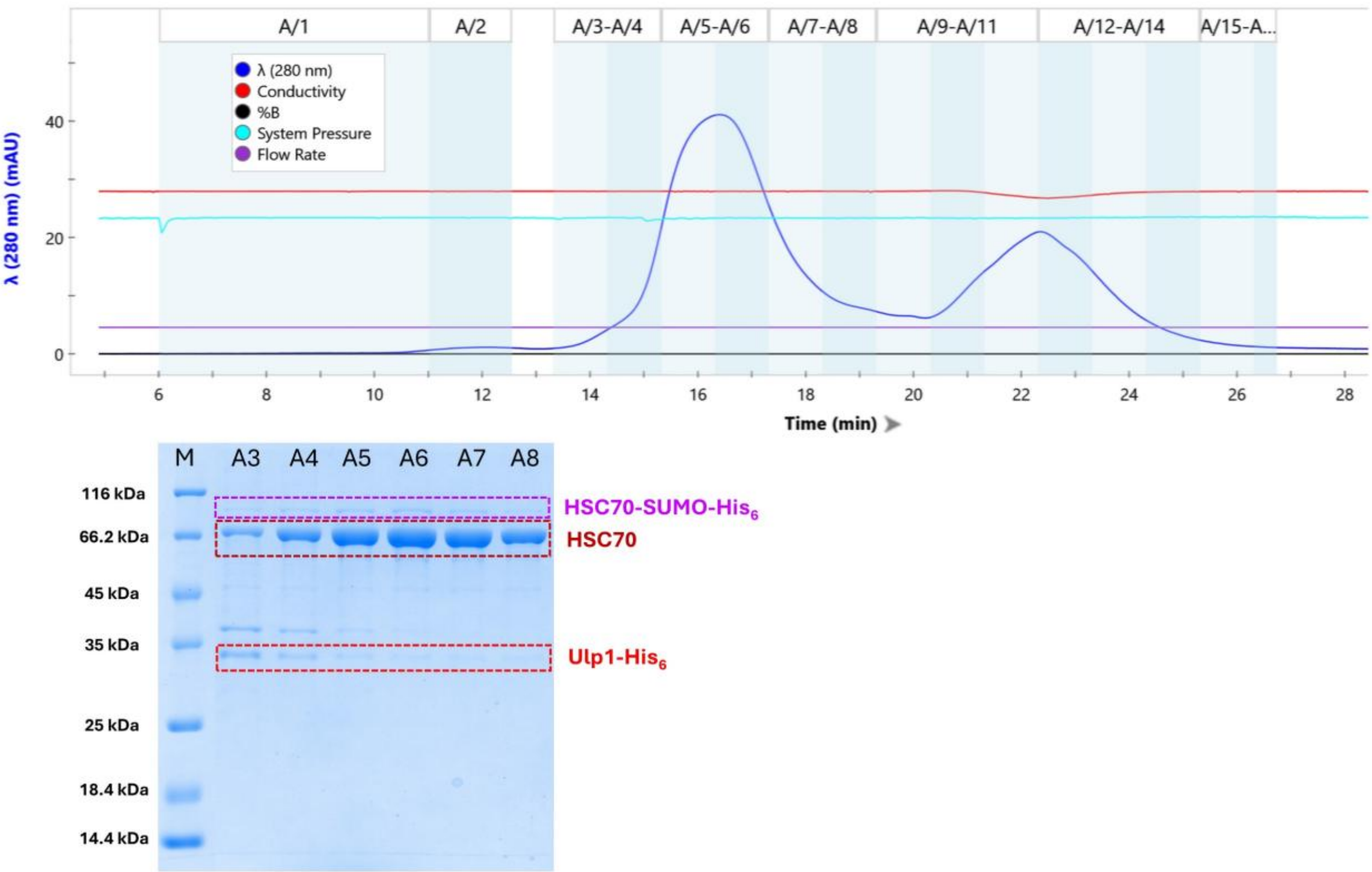

Figure S1. An exemplary SEC chromatogram of final purification step for ^{14}N-Hsc70 WT (**A**) and the corresponding SDS-PAGE analysis of the selected fractions (**B**). For all Hsc70 purifications, the most abundant fractions corresponding to Hsc70 monomer were collected and used for the following experiments and long-term storage (in this case, **A5-A8**). The early eluting fractions corresponding to oligomers/aggregates (**A2-A4**) were discarded. The second major peak assumingly corresponds to ATP (**A9-A14**). The gel analysis of fractions A3-A8 confirmed the majority of untagged Hsc70 (brown), with little contamination of Hsc70-SUMO-His_6 (magenta) and Ulp1-His_6 protease used for tag removal (red).

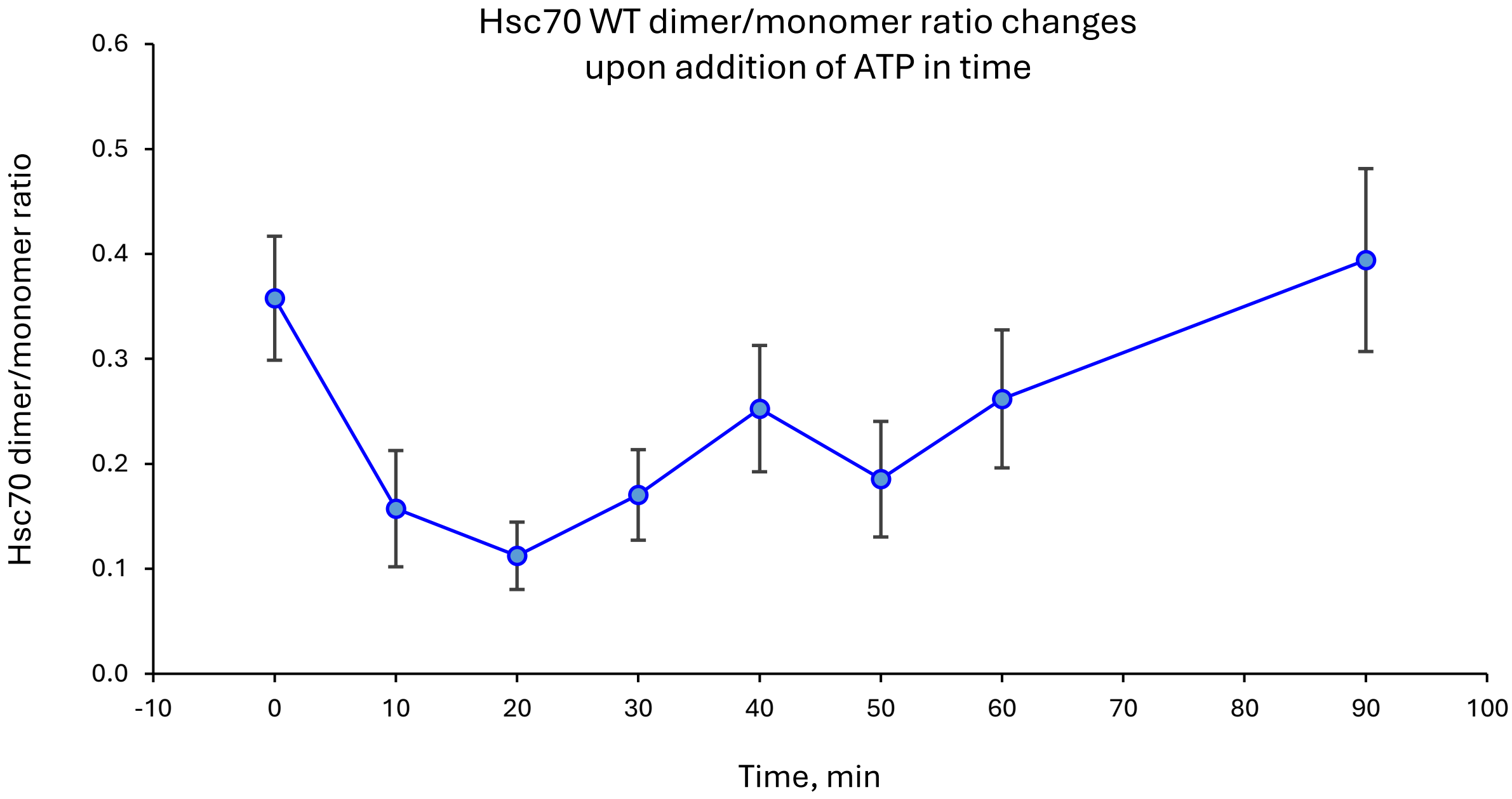

Figure S2. Time course of the Hsc70 WT dimer/monomer ratio after addition of ATP, analyzed by mass photometry. During the initial 20 min, the dimer level decreases to almost 1/3rd and starts to increase up to 90 min of incubation. Error bars represent standard deviation from the triplicate measurements (biological replicates).

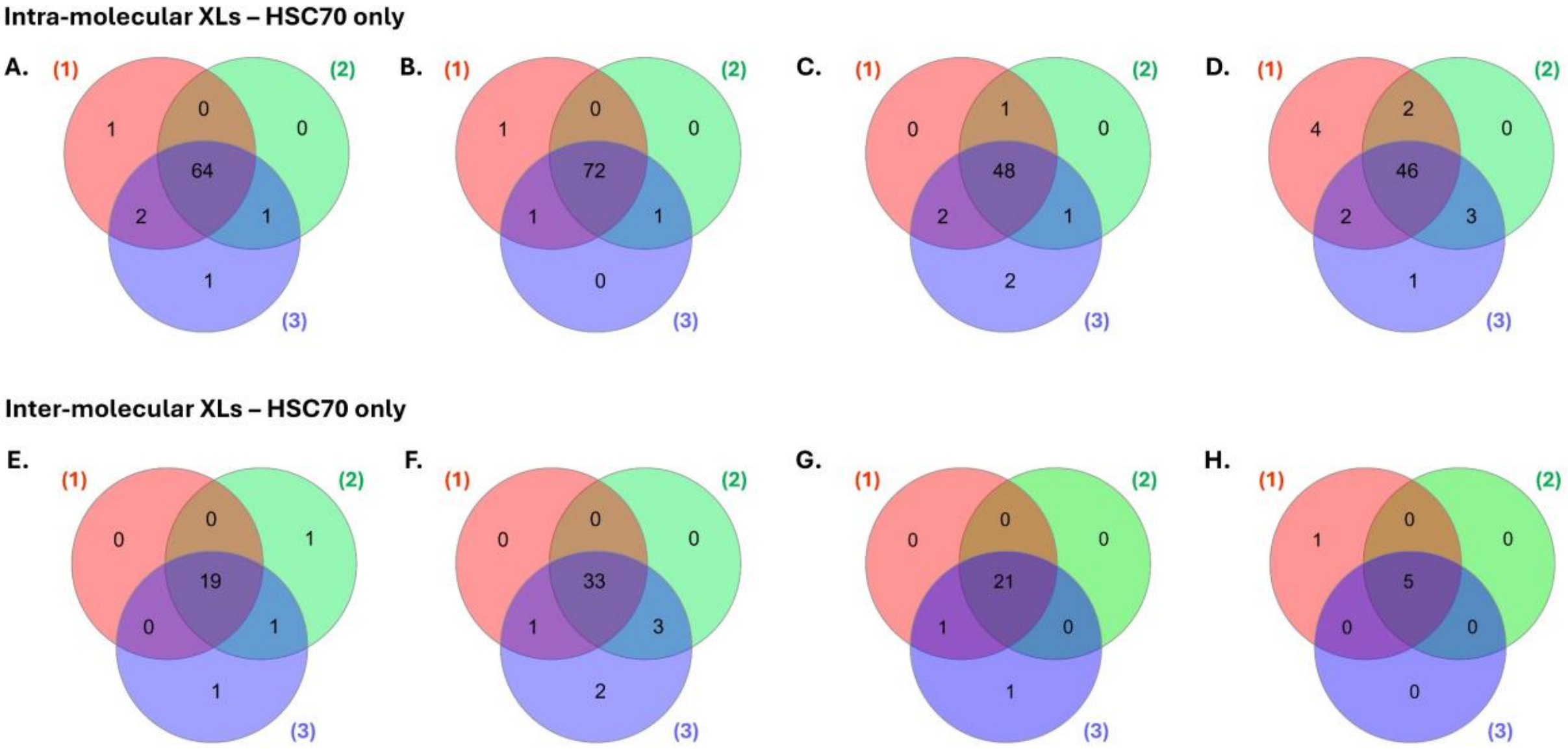

Figure S3. Venn diagrams of replicate intercept for Hsc70-only XL-MS experiments. **Top**: identified intra-XLs for ADP-state without dilution (**A**), ATP-state without dilution (**B**), ADP-state with dilution (**C**) and ATP-state with dilution (**D**). **Bottom:** identified inter-XLs for ADP-state without dilution (**E**), ATP-state without dilution (**F**), ADP-state with dilution (**G**) and ATP-state with dilution (**H**).

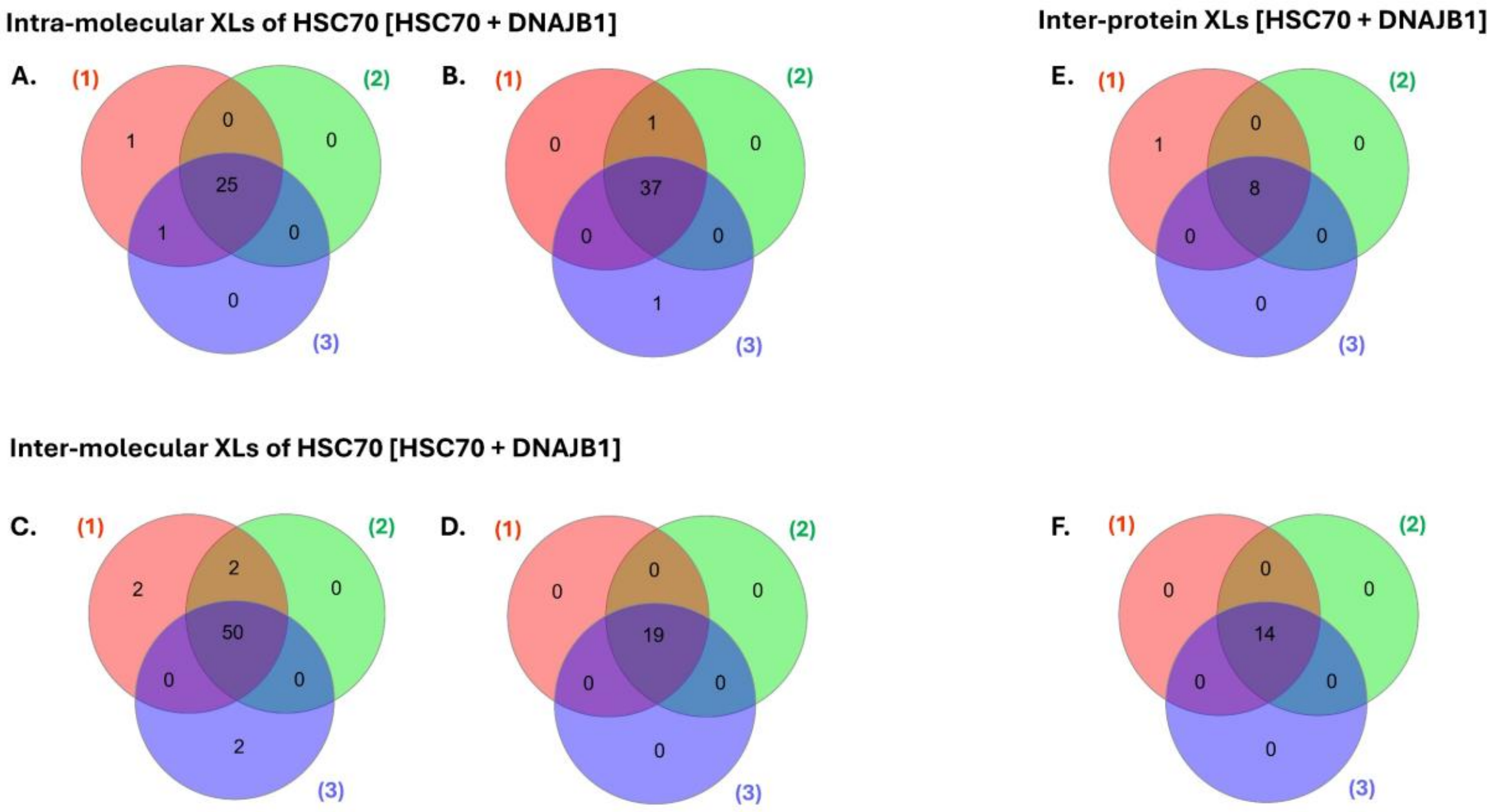

Figure S4. Venn diagrams of replicate intercept for Hsc70-DnaJB1 XL-MS experiments. **Top left**: identified Hsc70 intra-XLs with low DnaJB1(**A**) and high DnaJB1 (**B**). **Bottom left**: identified Hsc70 inter-XLs with low DnaJB1 (**C**) and high DnaJB1 (**D**). **Right:** identified Hsc70-DnaJB1 inter-XLs for low DnaJB1 (**E**) and high DnaJB1 (**F**).

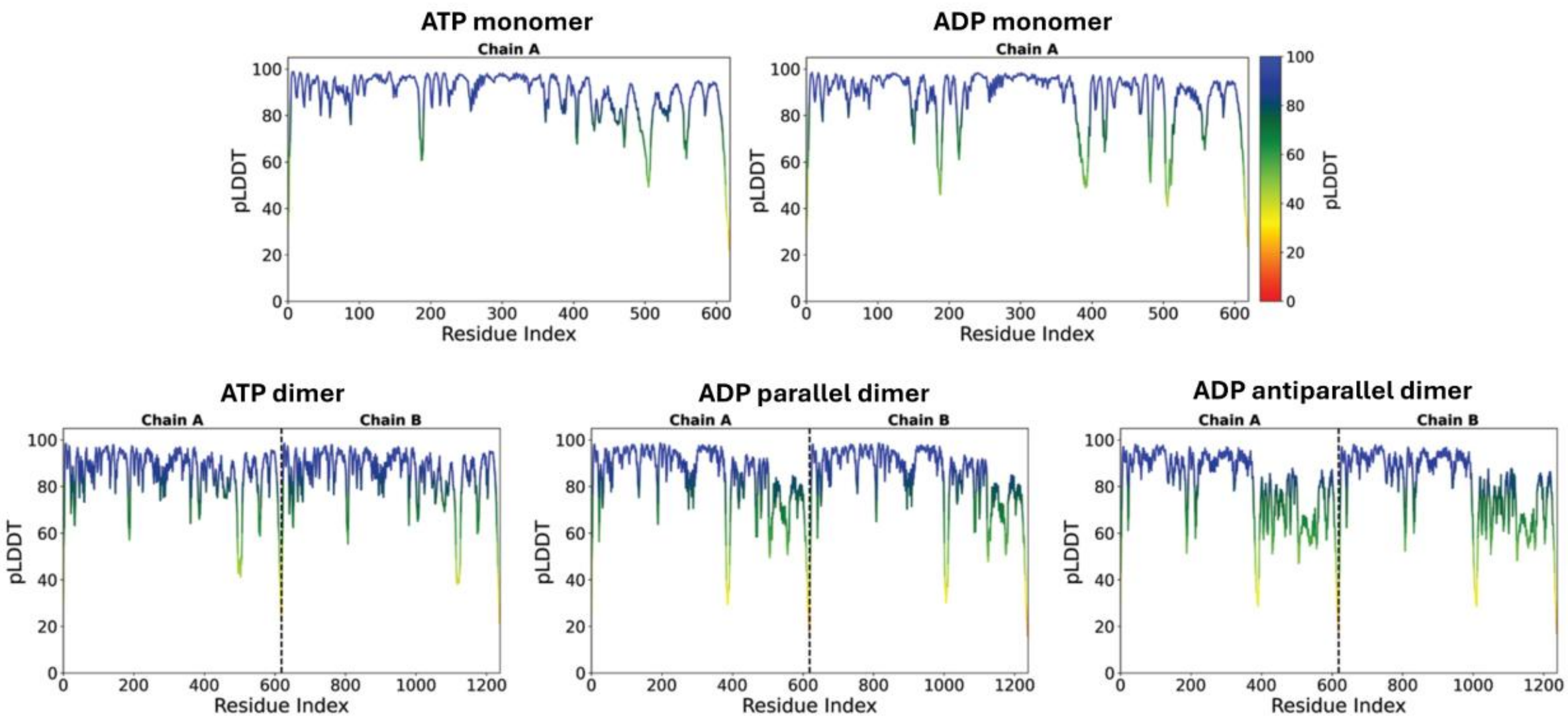

Figure S5. Prediction scores per residue (pLDDT) of the predicted Hsc70 models.

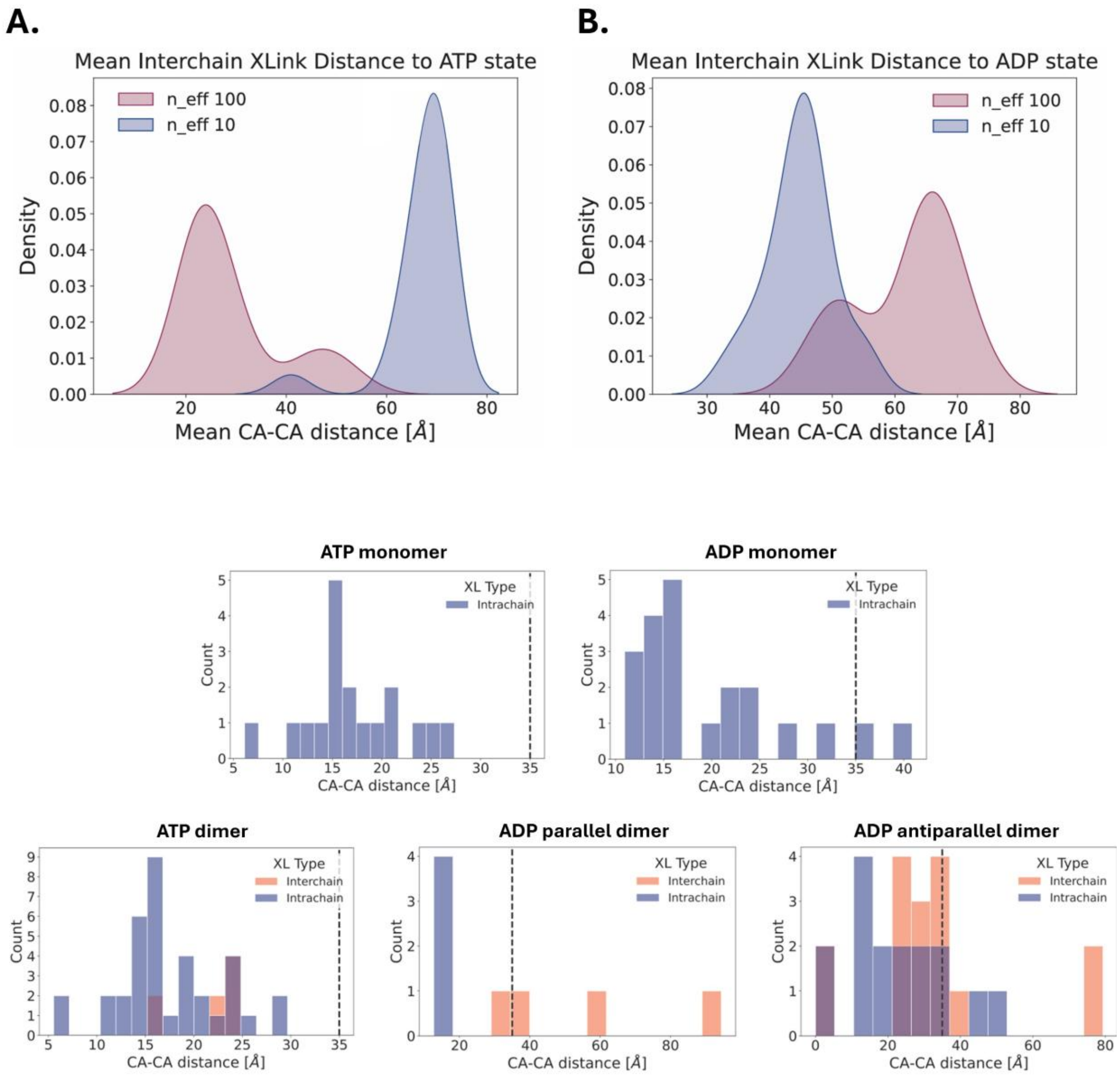

Figure S6. Distance constraint distributions of the predicted Hsc70 models.

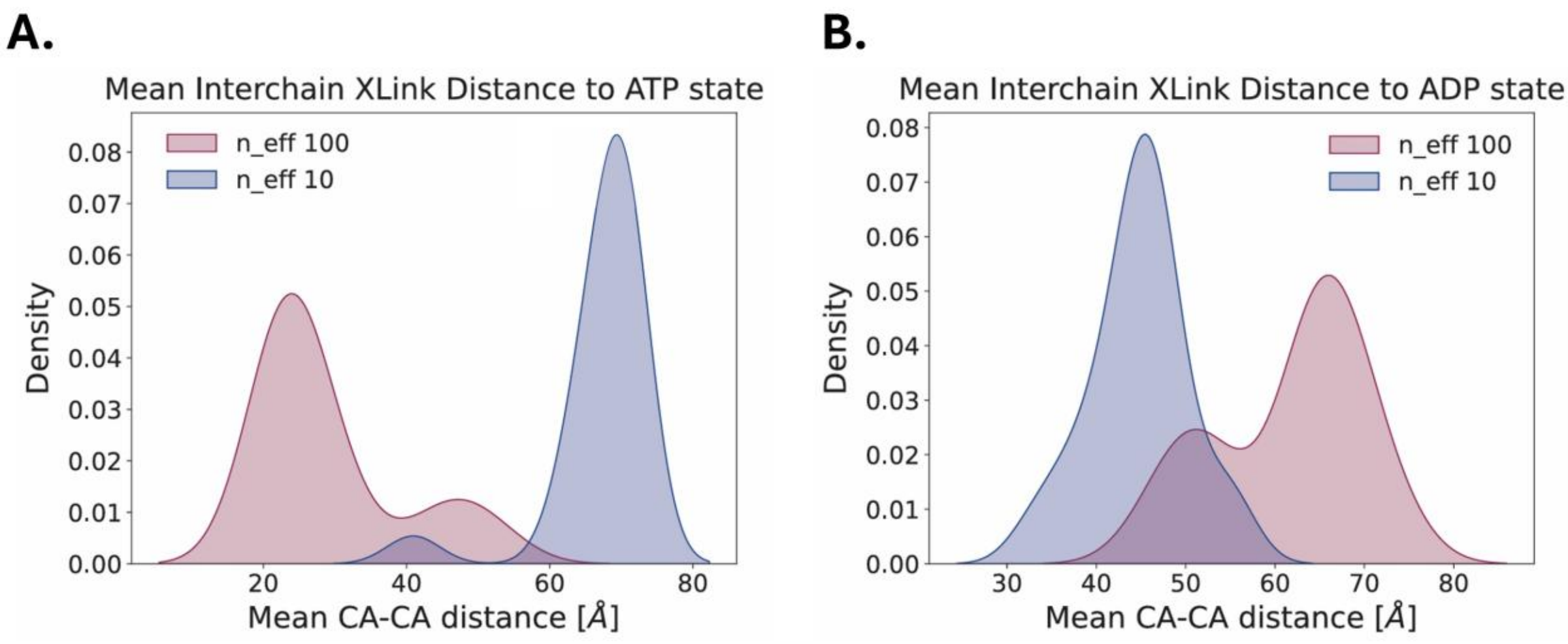

Figure S7. Effect of MSA subsampling (n_eff) on the Hsc70 dimer state. Models were generated with ADP-derived crosslink restraints; for each model we computed the mean Cα–Cα distances for the crosslinked residue pairs and compared them to ATP- and ADP reference crosslink distances. With less aggressive MSA subsampling (n_eff = 100), AlphaLink2 tends to produce ATP-like dimers (A, B). With more aggressive subsampling (n_eff = 10), models shift away from ATP and towards ADP (A, B).

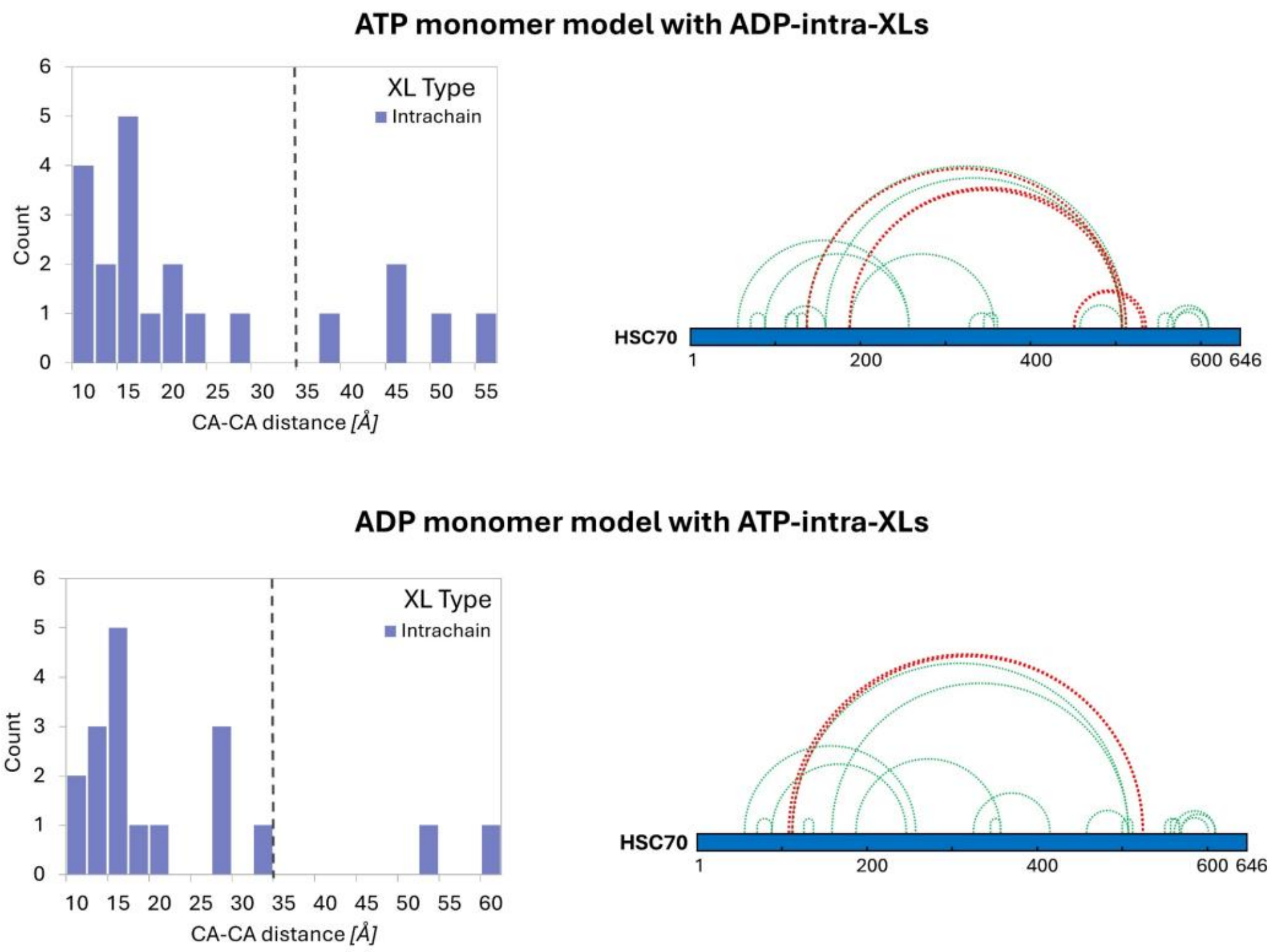

Figure S8. Analysis of distances for permutated XL sets. **Top** – analysis of XLs used for the ADP-Hsc70 monomer modelling on the predicted ATP-Hsc70 monomer model. **Bottom** – analysis of XLs used for the ATP-Hsc70 monomer modelling on the predicted ADP-Hsc70 monomer model.

Table S1. Total list of XLs identified in triplicates of the ATP-state and ADP-state experiments (with and without dilution) and their corresponding quantification (relative occurrence, inter/intra ratio); the list or residues utilized for the Hsc70 monomer and dimer modelling.

Unique Intra-XL			
**ATP-state, no dilution**	**ATP-state, with dilution**	**ADP-state, no dilution**	**ADP-state, with dilution**
K71-K187	K108-K535	K71-K159	K102-K531
K507-K526	Nterm-K550	K108-K159	K159-K524
K137-K458	K126-K535	Nterm-K512	K112-K497
K573-K589	K500-K567	K451-K539	K573-K601
K526-K539	K126-K526	K71-K137	K3-K512
K187-K246	K423-K497	K423-K557	K423-K500
K531-K550	K567-K609	K159-K257	
K126-K345	Nterm-K384	K126-K159	
K251-K561		K512-K524	
K497-K512		Nterm-K531	
K325-K357		K159-K188	
K88-K112		K220-K325	
K126-K319		K25-K188	
K512-K539		K187-K345	
K112-K128		K319-K415	
K71-K112		Nterm-K415	
K15-9K246		K102-K112	
K415-K512		K71-K108	
K507-K539			
K539-K557			
K108-K531			
K557-K573			
K325-K345			
K451-K500			
K126-K188			
K246-K271			
K56-K102			
K71-K188			
K524-K535			
K246-K319			
K573-K597			
K271-K319			

Ambiguous Intra-XL							
**XL**	**Experiment**	**XL relative occurrence**	**XL relative occurrence SD**	**XL**	**Experiment**	**XL relative occurrence**	**XL relative occurrence SD**
K328-K357	ATP-state, no dilution	0.00504	0.00016	K108-K524	ATP-state, no dilution	0.00000	0.00000
	ATP-state, with dilution	0.00303	0.00038		ATP-state, with dilution	0.00655	0.00045
	ADP-state, no dilution	0.00416	0.00121		ADP-state, no dilution	0.00412	0.00114
	ADP-state, with dilution	0.00699	0.00021		ADP-state, with dilution	0.00242	0.00030
K569-K609	ATP-state, no dilution	0.00996	0.00073	K112-K507	ATP-state, no dilution	0.00000	0.00000
	ATP-state, with dilution	0.00484	0.00075		ATP-state, with dilution	0.01090	0.00134
	ADP-state, no dilution	0.01084	0.00057		ADP-state, no dilution	0.00742	0.00138
	ADP-state, with dilution	0.00644	0.00107		ADP-state, with dilution	0.00000	0.00000
K500-K512	ATP-state, no dilution	0.02071	0.00097	K188-K357	ATP-state, no dilution	0.00000	0.00000
	ATP-state, with dilution	0.01418	0.00039		ATP-state, with dilution	0.00317	0.00053
	ADP-state, no dilution	0.00949	0.00136		ADP-state, no dilution	0.00461	0.00087
	ADP-state, with dilution	0.00373	0.00043		ADP-state, with dilution	0.00534	0.00084
Nterm-K159	ATP-state, no dilution	0.01806	0.00051	K137-K507	ATP-state, no dilution	0.00155	0.00006
	ATP-state, with dilution	0.01631	0.00245		ATP-state, with dilution	0.00238	0.00050
	ADP-state, no dilution	0.03183	0.00516		ADP-state, no dilution	0.00453	0.00096
	ADP-state, with dilution	0.02360	0.00479		ADP-state, with dilution	0.00661	0.00093
K159-K507	ATP-state, no dilution	0.00607	0.00027	K112-K524	ATP-state, no dilution	0.01286	0.00080
	ATP-state, with dilution	0.00348	0.00027		ATP-state, with dilution	0.01342	0.00126
	ADP-state, no dilution	0.00889	0.00091		ADP-state, no dilution	0.00912	0.00175
	ADP-state, with dilution	0.00609	0.00016		ADP-state, with dilution	0.00000	0.00000
K88-K257	ATP-state, no dilution	0.00796	0.00042	K561-K609	ATP-state, no dilution	0.00612	0.00109
	ATP-state, with dilution	0.00106	0.00018		ATP-state, with dilution	0.00263	0.00134
	ADP-state, no dilution	0.01177	0.00110		ADP-state, no dilution	0.00000	0.00000
	ADP-state, with dilution	0.00270	0.00047		ADP-state, with dilution	0.00289	0.00030

XL	Experiment	XL relative occurrence	XL relative occurrence SD	XL	Experiment	XL relative occurrence	XL relative occurrence SD
K451-K531	ATP-state, no dilution	0.00240	0.00027	K325-K415	ATP-state, no dilution	0.05678	0.00302
	ATP-state, with dilution	0.00125	0.00016		ATP-state, with dilution	0.05453	0.00947
	ADP-state, no dilution	0.00457	0.00094		ADP-state, no dilution	0.00000	0.00000
	ADP-state, with dilution	0.00373	0.00059		ADP-state, with dilution	0.02368	0.00358
K71-K88	ATP-state, no dilution	0.00731	0.00011	K159-K497	ATP-state, no dilution	0.00173	0.00022
	ATP-state, with dilution	0.00105	0.00027		ATP-state, with dilution	0.00098	0.00014
	ADP-state, no dilution	0.00387	0.00077		ADP-state, no dilution	0.00000	0.00000
	ADP-state, with dilution	0.00101	0.00022		ADP-state, with dilution	0.00194	0.00036
K112-K159	ATP-state, no dilution	0.00384	0.00039	K56-K257	ATP-state, no dilution	0.00312	0.00011
	ATP-state, with dilution	0.00195	0.00018		ATP-state, with dilution	0.00171	0.00037
	ADP-state, no dilution	0.00475	0.00047		ADP-state, no dilution	0.00000	0.00000
	ADP-state, with dilution	0.00285	0.00024		ADP-state, with dilution	0.00235	0.00004
K345-K357	ATP-state, no dilution	0.00915	0.00018	K159-K187	ATP-state, no dilution	0.00000	0.00000
	ATP-state, with dilution	0.00317	0.00079		ATP-state, with dilution	0.00000	0.00000
	ADP-state, no dilution	0.00581	0.00046		ADP-state, no dilution	0.00427	0.00114
	ADP-state, with dilution	0.00245	0.00050		ADP-state, with dilution	0.00087	0.00016
K569-K601	ATP-state, no dilution	0.00531	0.00014	K187-K512	ATP-state, no dilution	0.00000	0.00000
	ATP-state, with dilution	0.00284	0.00078		ATP-state, with dilution	0.00000	0.00000
	ADP-state, no dilution	0.00698	0.00173		ADP-state, no dilution	0.00382	0.00033
	ADP-state, with dilution	0.00334	0.00039		ADP-state, with dilution	0.00301	0.00002
Nterm-K137	ATP-state, no dilution	0.01224	0.00048	K112-K126	ATP-state, no dilution	0.00000	0.00000
	ATP-state, with dilution	0.01580	0.00237		ATP-state, with dilution	0.00000	0.00000
	ADP-state, no dilution	0.01466	0.00219		ADP-state, no dilution	0.00802	0.00181
	ADP-state, with dilution	0.01693	0.00147		ADP-state, with dilution	0.00167	0.00015
K458-K507	ATP-state, no dilution	0.02330	0.00334	K187-K507	ATP-state, no dilution	0.00000	0.00000
	ATP-state, with dilution	0.03209	0.00459		ATP-state, with dilution	0.00000	0.00000
	ADP-state, no dilution	0.03368	0.00253		ADP-state, no dilution	0.00335	0.00034
	ADP-state, with dilution	0.03653	0.00061		ADP-state, with dilution	0.00374	0.00052

XL	Experiment	XL relative occurrence	XL relative occurrence SD	XL	Experiment	XL relative occurrence	XL relative occurrence SD
K88-K246	ATP-state, no dilution	0.03889	0.00081	K361-K524	ATP-state, no dilution	0.00000	0.00000
	ATP-state, with dilution	0.04337	0.00905		ATP-state, with dilution	0.00000	0.00000
	ADP-state, no dilution	0.01081	0.00235		ADP-state, no dilution	0.00140	0.00028
	ADP-state, with dilution	0.01325	0.00150		ADP-state, with dilution	0.00216	0.00019
K451-K535	ATP-state, no dilution	0.00605	0.00067	K187-K328	ATP-state, no dilution	0.00808	0.00003
	ATP-state, with dilution	0.00507	0.00064		ATP-state, with dilution	0.00000	0.00000
	ADP-state, no dilution	0.01090	0.00193		ADP-state, no dilution	0.00275	0.00044
	ADP-state, with dilution	0.01343	0.00044		ADP-state, with dilution	0.00000	0.00000
K526-K561	ATP-state, no dilution	0.00109	0.00015	K108-K126	ATP-state, no dilution	0.00771	0.00042
	ATP-state, with dilution	0.00146	0.00009		ATP-state, with dilution	0.00000	0.00000
	ADP-state, no dilution	0.00136	0.00025		ADP-state, no dilution	0.00500	0.00102
	ADP-state, with dilution	0.00139	0.00011		ADP-state, with dilution	0.00000	0.00000
K550-K567	ATP-state, no dilution	0.00854	0.00106	K328-K345	ATP-state, no dilution	0.00433	0.00093
	ATP-state, with dilution	0.01139	0.00178		ATP-state, with dilution	0.00000	0.00000
	ADP-state, no dilution	0.00758	0.00110		ADP-state, no dilution	0.00176	0.00009
	ADP-state, with dilution	0.01137	0.00082		ADP-state, with dilution	0.00000	0.00000
K137-K512	ATP-state, no dilution	0.00141	0.00014	K56-K71	ATP-state, no dilution	0.03070	0.00389
	ATP-state, with dilution	0.00245	0.00038		ATP-state, with dilution	0.00000	0.00000
	ADP-state, no dilution	0.00596	0.00104		ADP-state, no dilution	0.01460	0.00521
	ADP-state, with dilution	0.00733	0.00056		ADP-state, with dilution	0.00000	0.00000
K126-K137	ATP-state, no dilution	0.00836	0.00100	K88-K102	ATP-state, no dilution	0.00483	0.00064
	ATP-state, with dilution	0.00178	0.00068		ATP-state, with dilution	0.00000	0.00000
	ADP-state, no dilution	0.00605	0.00052		ADP-state, no dilution	0.00365	0.00085
	ADP-state, with dilution	0.00187	0.00033		ADP-state, with dilution	0.00000	0.00000

XL	Experiment	XL relative occurrence	XL relative occurrence SD	XL	Experiment	XL relative occurrence	XL relative occurrence SD
K187-K357	ATP-state, no dilution	0.00385	0.00016	K526-K573	ATP-state, no dilution	0.00572	0.00001
	ATP-state, with dilution	0.00483	0.00110		ATP-state, with dilution	0.00000	0.00000
	ADP-state, no dilution	0.00184	0.00032		ADP-state, no dilution	0.00747	0.00121
	ADP-state, with dilution	0.00151	0.00015		ADP-state, with dilution	0.00000	0.00000
K557-K601	ATP-state, no dilution	0.00175	0.00018	K535-K550	ATP-state, no dilution	0.00611	0.00023
	ATP-state, with dilution	0.00000	0.00000		ATP-state, with dilution	0.00000	0.00000
	ADP-state, no dilution	0.00185	0.00040		ADP-state, no dilution	0.00286	0.00071
	ADP-state, with dilution	0.00080	0.00008		ADP-state, with dilution	0.00000	0.00000
K88-K126	ATP-state, no dilution	0.00269	0.00035	K137-K159	ATP-state, no dilution	0.00313	0.00033
	ATP-state, with dilution	0.00000	0.00000		ATP-state, with dilution	0.00000	0.00000
	ADP-state, no dilution	0.00184	0.00036		ADP-state, no dilution	0.00248	0.00040
	ADP-state, with dilution	0.00096	0.00007		ADP-state, with dilution	0.00000	0.00000
K345-K361	ATP-state, no dilution	0.00455	0.00016	K539-K550	ATP-state, no dilution	0.01391	0.00072
	ATP-state, with dilution	0.00000	0.00000		ATP-state, with dilution	0.00000	0.00000
	ADP-state, no dilution	0.00233	0.00031		ADP-state, no dilution	0.00431	0.00146
	ADP-state, with dilution	0.00186	0.00038		ADP-state, with dilution	0.00000	0.00000
K108-K271	ATP-state, no dilution	0.00000	0.00000	Nterm-K25	ATP-state, no dilution	0.00000	0.00000
	ATP-state, with dilution	0.00089	0.00001		ATP-state, with dilution	0.00142	0.00033
	ADP-state, no dilution	0.00165	0.00021		ADP-state, no dilution	0.00000	0.00000
	ADP-state, with dilution	0.00150	0.00023		ADP-state, with dilution	0.00125	0.00022
Nterm-K507	ATP-state, no dilution	0.00000	0.00000	K557-K567	ATP-state, no dilution	0.00382	0.00025
	ATP-state, with dilution	0.00287	0.00013		ATP-state, with dilution	0.00370	0.00273
	ADP-state, no dilution	0.00279	0.00055		ADP-state, no dilution	0.00000	0.00000
	ADP-state, with dilution	0.00679	0.00013		ADP-state, with dilution	0.00000	0.00000
K250-K500	ATP-state, no dilution	0.00000	0.00000	Nterm-K458	ATP-state, no dilution	0.00179	0.00011
	ATP-state, with dilution	0.00123	0.00003		ATP-state, with dilution	0.00175	0.00071
	ADP-state, no dilution	0.00085	0.00018		ADP-state, no dilution	0.00000	0.00000
	ADP-state, with dilution	0.00121	0.00013		ADP-state, with dilution	0.00000	0.00000

XL	Experiment	XL relative occurrence	XL relative occurrence SD	XL	Experiment	XL relative occurrence	XL relative occurrence SD
Nterm-K187	ATP-state, no dilution	0.00000	0.00000	K3-K187	ATP-state, no dilution	0.00000	0.00000
	ATP-state, with dilution	0.00212	0.00038		ATP-state, with dilution	0.00212	0.00038
	ADP-state, no dilution	0.00130	0.00029		ADP-state, no dilution	0.00130	0.00029
	ADP-state, with dilution	0.00339	0.00045		ADP-state, with dilution	0.00339	0.00045
K108-K524	ATP-state, no dilution	0.00000	0.00000	K3-K137	ATP-state, no dilution	0.00000	0.00000
	ATP-state, with dilution	0.00655	0.00045		ATP-state, with dilution	0.00400	0.00080
	ADP-state, no dilution	0.00412	0.00114		ADP-state, no dilution	0.00000	0.00000
	ADP-state, with dilution	0.00242	0.00030		ADP-state, with dilution	0.00376	0.00027

Unique Inter-XL

ATP-state, no dilution			ATP-state, with dilution			ADP-state, no dilution			ADP-state, with dilution		
XL	Inter/intra ratio	Inter/intra ratio SD	XL	Inter/intra ratio	Inter/intra ratio SD	XL	Inter/intra ratio	Inter/intra ratio SD	XL	Inter/intra ratio	Inter/intra ratio SD
K159-K271	0.216	0.002				K56-K271	0.231	0.026	Nterm-K524	0.089	0.007
K451-K512	0.122	0.003				K159-K451	0.283	0.012	K345-K524	0.514	0.023
K71-K250	0.283	0.018				K187-K319	0.243	0.009	K345-K597	0.436	0.044
K451-K507	0.157	0.012							Nterm-K458	0.255	0.012
K159-K188	0.102	0.013							K507-K597	0.320	0.021
K159-K325	0.255	0.007							K188-K458	0.463	0.024
K159-K257	0.189	0.086							K159-K458	0.316	0.011
K187-K345	0.288	0.033							K187-K458	0.379	0.008
K71-K257	0.221	0.022							K137-K524	0.113	0.002
K71-K507	0.328	0.034							K423-K557	0.344	0.021
K159-K319	0.263	0.016							K137-K583	0.350	0.028
K56-K325	0.337	0.008							K137-K550	0.467	0.029
K71-K451	0.321	0.016									
K159-K187	0.135	0.018									
K71-K159	0.255	0.025									
K126-K187	0.318	0.011									
K271-K423	0.196	0.017									
K56-K187	0.263	0.005									
K137-K319	0.263	0.024									
K56-K328	0.327	0.022									

Ambiguous Inter-XL					
XL	Experiment	XL relative occurrence	XL relative occurrence SD	Inter/intra ratio	Inter/intra ratio SD
K345-K557	ATP-state, no dilution	0.00118	0.00013	0.914	0.027
	ATP-state, with dilution	0.00118	0.00012	0.886	0.046
	ADP-state, no dilution	0.00625	0.00206	0.935	0.030
	ADP-state, with dilution	0.00079	0.00010	0.827	0.046
K137-K188	ATP-state, no dilution	0.00399	0.00001	0.553	0.011
	ATP-state, with dilution	0.00273	0.00031	0.826	0.021
	ADP-state, no dilution	0.00660	0.00243	0.764	0.051
	ADP-state, with dilution	0.00204	0.00040	0.758	0.024
K137-K187	ATP-state, no dilution	0.00675	0.00007	0.644	0.006
	ATP-state, with dilution	0.00409	0.00089	0.879	0.025
	ADP-state, no dilution	0.00659	0.00077	0.782	0.029
	ADP-state, with dilution	0.00241	0.00051	0.749	0.052
K328-K557	ATP-state, no dilution	0.00174	0.00016	0.987	0.021
	ATP-state, with dilution	0.00257	0.00031	1.003	0.046
	ADP-state, no dilution	0.00233	0.00090	0.963	0.022
	ADP-state, with dilution	0.00133	0.00014	0.970	0.041
K159-K345	ATP-state, no dilution	0.00305	0.00015	0.312	0.015
	ATP-state, with dilution	0.00000	0.00000	0.000	0.000
	ADP-state, no dilution	0.00368	0.00084	0.554	0.038
	ADP-state, with dilution	0.00181	0.00006	0.846	0.017

XL	Experiment	XL relative occurrence	XL relative occurrence SD	Inter/intra ratio	Inter/intra ratio SD
K500-K561	ATP-state, no dilution	0.00127	0.00007	0.089	0.023
	ATP-state, with dilution	0.00000	0.00000	0.000	0.000
	ADP-state, no dilution	0.00212	0.00033	0.267	0.033
	ADP-state, with dilution	0.00216	0.00030	0.350	0.043
K250-K583	ATP-state, no dilution	0.00083	0.00009	0.909	0.046
	ATP-state, with dilution	0.00179	0.00029	0.963	0.021
	ADP-state, no dilution	0.00090	0.00023	0.855	0.164
	ADP-state, with dilution	0.00000	0.00000	0.000	0.000
K415-K512	ATP-state, no dilution	0.00000	0.00000	0.000	0.000
	ATP-state, with dilution	0.00000	0.00000	0.000	0.000
	ADP-state, no dilution	0.00752	0.00066	0.229	0.005
	ADP-state, with dilution	0.00381	0.00044	0.314	0.003
K137-K458	ATP-state, no dilution	0.00000	0.00000	0.000	0.000
	ATP-state, with dilution	0.00000	0.00000	0.000	0.000
	ADP-state, no dilution	0.00185	0.00024	0.408	0.027
	ADP-state, with dilution	0.00237	0.00016	0.446	0.007
K137-K497	ATP-state, no dilution	0.00000	0.00000	0.000	0.000
	ATP-state, with dilution	0.00000	0.00000	0.000	0.000
	ADP-state, no dilution	0.00138	0.00025	0.341	0.035
	ADP-state, with dilution	0.00126	0.00009	0.422	0.003

XL	Experiment	XL relative occurrence	XL relative occurrence SD	Inter/intra ratio	Inter/intra ratio SD
K71-K319	ATP-state, no dilution	0.00272	0.00008	0.285	0.012
	ATP-state, with dilution	0.00000	0.00000	0.000	0.000
	ADP-state, no dilution	0.00130	0.00025	0.323	0.027
	ADP-state, with dilution	0.00000	0.00000	0.000	0.000
K187-K271	ATP-state, no dilution	0.00310	0.00015	0.190	0.011
	ATP-state, with dilution	0.00000	0.00000	0.000	0.000
	ADP-state, no dilution	0.00183	0.00044	0.215	0.032
	ADP-state, with dilution	0.00000	0.00000	0.000	0.000
K137-K271	ATP-state, no dilution	0.00253	0.00013	0.224	0.016
	ATP-state, with dilution	0.00000	0.00000	0.000	0.000
	ADP-state, no dilution	0.00168	0.00029	0.269	0.046
	ADP-state, with dilution	0.00000	0.00000	0.000	0.000
Nterm-K187	ATP-state, no dilution	0.00321	0.00007	0.219	0.012
	ATP-state, with dilution	0.00000	0.00000	0.000	0.000
	ADP-state, no dilution	0.00710	0.00200	0.151	0.029
	ADP-state, with dilution	0.00000	0.00000	0.000	0.000
K126-K361	ATP-state, no dilution	0.00101	0.00023	0.815	0.040
	ATP-state, with dilution	0.00000	0.00000	0.000	0.000
	ADP-state, no dilution	0.00146	0.00023	0.853	0.047
	ADP-state, with dilution	0.00000	0.00000	0.000	0.000

XL	Experiment	XL relative occurrence	XL relative occurrence SD	Inter/intra ratio	Inter/intra ratio SD
K71-K271	ATP-state, no dilution	0.00354	0.00010	0.255	0.011
	ATP-state, with dilution	0.00000	0.00000	0.000	0.000
	ADP-state, no dilution	0.00174	0.00067	0.290	0.019
	ADP-state, with dilution	0.00000	0.00000	0.000	0.000

Monomer modelling		Dimer modelling		
ATP-state	ADP-state	ATP-state	ADP-state (antiparallel)	ADP-state (parallel)
A.569-A.609	A.328-A.357	A.569-A.609	A.451-A.531	A.451-A.531
A.500-A.512	A.569-A.609	A.500-A.512	A.451-A.535	A.451-A.535
A.159-A.507	A.159-A.507	A.159-A.507	A.137-A.512	B.451-B.531
A.71-A.88	A.88-A.257	A.71-A.88	A.1-A.507	B.451-B.535
A.345-A.357	A.451-A.531	A.345-A.357	A.137-A.507	A.500-B.561
A.569-A.601	A.71-A.88	A.569-A.601	A.187-A.512	A.512-B.415
A.458-A.507	A.112-A.159	A.458-A.507	A.187-A.507	A.137-B.458
A.88-A.246	A.569-A.601	A.88-A.246	A.3-A.187	A.137-B.497
A.550-A.567	A.458-A.507	A.550-A.567	B.451-B.531	
A.126-A.137	A.451-A.535	A.126-A.137	B.451-B.535	
A.187-A.357	A.550-A.567	A.187-A.357	B.137-B.512	
A.108-A.524	A.137-A.512	A.108-A.524	B.1-B.507	
A.112-A.507	A.126-A.137	A.112-A.507	B.137-B.507	
A.112-A.524	A.345-A.361	A.112-A.524	B.187-B.512	
A.561-A.609	A.188-A.357	A.561-A.609	B.187-B.507	
A.325-A.415	A.137-A.507	A.325-A.415	B.3-B.187	
A.56-A.257	A.561-A.609	A.56-A.257	A.415-B.512	
A.557-A.567	A.56-A.257	A.557-A.567	B.415-A.512	
	A.187-A.512	B.569-B.609	A.137-B.458	
	A.112-A.126	B.500-B.512	B.137-A.458	
	A.187-A.507	B.159-B.507	A.137-B.497	
		B.71-B.88	B.137-A.497	
		B.345-B.357	A.345-B.597	
		B.569-B.601	B.345-A.597	
		B.458-B.507	A.1-B.458	
		B.88-B.246	B.1-A.458	
		B.550-B.567	A.188-B.458	
		B.126-B.137	B.188-A.458	
		B.187-B.357	A.137-B.583	
		B.108-B.524	B.137-A.583	
		B.112-B.507	A.137-B.550	
		B.112-B.524	B.137-A.550	
		B.561-B.609		
		B.325-B.415		
		B.56-B.257		
		B.557-B.567		
		A.345-B.557		
		A.557-B.345		
		A.137-B.188		
		A.188-B.137		
		A.328-B.557		
		A.557-B.328		
		A.250-B.583		
		A.583-B.250		

Table S2. Total list of XLs identified in triplicates of the ATP-state, ADP-state, low-DNAJB1 and high-DNAJB1 experiments (without dilution) and their corresponding quantification (relative occurrence, inter/intra ratio).

**Unique Intra-XL**			
**ATP-state, no dilution**	**ADP-state, no dilution**	**low-DNAJB1**	**high-DNAJB1**
K159-K497	K71-K159	K188-K524	K159-K458
K71-K187	K108-K159	K500-K531	
K507-K526	K451-K539	K348-K524	
K137-K458	K71-K137	K159-K526	
K526-K539	K423-K557	K3-K512	
K187-K246	K159-K257	K112-K526	
K531-K550	K126-K159	K3-K159	
K126-K345	K512-K524	K423-K500	
K251-K561	Nterm-K531		
K497-K512	K220-K325		
K325-K357	K25-K188		
K88-K112	K187-K345		
K126-K319	K319-K415		
K512-K539	Nterm-K415		
K112-K128	K102-K112		
Nterm_K458	K361-K524		
K561-K609	K71-K108		
K71-K112			
K159-K246			
K415-K512			
K507-K539			
K539-K557			
K108-K531			
K557-K573			
K325-K345			
K451-K500			
K126-K188			
K246-K271			
K325-K415			
K56-K102			
K71-K188			
K524-K535			
K246-K319			
K573-K597			
K271-K319			

Ambiguous Intra-XL							
**XL**	**Experiment**	**XL relative occurrence**	**XL relative occurrence SD**	**XL**	**Experiment**	**XL relative occurrence**	**XL relative occurrence SD**
Nterm-K159	ATP-state, no dilution	0.01806	0.00051	K187-K512	ATP-state, no dilution	0.00000	0.00000
	ADP-state, no dilution	0.03183	0.00516		ADP-state, no dilution	0.00382	0.00033
	low-DNAJB1	0.02059	0.00321		low-DNAJB1	0.00319	0.00021
	high-DNAJB1	0.02723	0.00105		high-DNAJB1	0.00488	0.00004
K345-K357	ATP-state, no dilution	0.00915	0.00018	K112-K126	ATP-state, no dilution	0.00000	0.00000
	ADP-state, no dilution	0.00581	0.00046		ADP-state, no dilution	0.00802	0.00181
	low-DNAJB1	0.00552	0.00032		low-DNAJB1	0.00100	0.00012
	high-DNAJB1	0.00729	0.00098		high-DNAJB1	0.00156	0.00013
Nterm-K137	ATP-state, no dilution	0.01224	0.00048	K3-K187	ATP-state, no dilution	0.00000	0.00000
	ADP-state, no dilution	0.01466	0.00219		ADP-state, no dilution	0.00130	0.00029
	low-DNAJB1	0.00463	0.00024		low-DNAJB1	0.00078	0.00005
	high-DNAJB1	0.00891	0.00057		high-DNAJB1	0.00000	0.00000
K328-K357	ATP-state, no dilution	0.00504	0.00016	K108-K524	ATP-state, no dilution	0.00000	0.00000
	ADP-state, no dilution	0.00416	0.00121		ADP-state, no dilution	0.00412	0.00114
	low-DNAJB1	0.00294	0.00015		low-DNAJB1	0.00199	0.00018
	high-DNAJB1	0.00371	0.00022		high-DNAJB1	0.00192	0.00010
K159-K507	ATP-state, no dilution	0.00607	0.00027	K187-K507	ATP-state, no dilution	0.00000	0.00000
	ADP-state, no dilution	0.00889	0.00091		ADP-state, no dilution	0.00335	0.00034
	low-DNAJB1	0.00599	0.00075		low-DNAJB1	0.00312	0.00018
	high-DNAJB1	0.00810	0.00021		high-DNAJB1	0.00473	0.00015
K56-K71	ATP-state, no dilution	0.03070	0.00389	K188-K357	ATP-state, no dilution	0.00000	0.00000
	ADP-state, no dilution	0.01460	0.00521		ADP-state, no dilution	0.00461	0.00087
	low-DNAJB1	0.00210	0.00011		low-DNAJB1	0.00244	0.00011
	high-DNAJB1	0.00321	0.00013		high-DNAJB1	0.00288	0.00005

XL	Experiment	XL relative occurrence	XL relative occurrence SD	XL	Experiment	XL relative occurrence	XL relative occurrence SD
K88-K257	ATP-state, no dilution	0.00796	0.00042	K187-K328	ATP-state, no dilution	0.00808	0.00003
	ADP-state, no dilution	0.01177	0.00110		ADP-state, no dilution	0.00275	0.00044
	low-DNAJB1	0.00892	0.00185		low-DNAJB1	0.00000	0.00000
	high-DNAJB1	0.01211	0.00015		high-DNAJB1	0.00000	0.00000
K451-K531	ATP-state, no dilution	0.00240	0.00027	K108K126	ATP-state, no dilution	0.00771	0.00042
	ADP-state, no dilution	0.00457	0.00094		ADP-state, no dilution	0.00500	0.00102
	low-DNAJB1	0.00149	0.00013		low-DNAJB1	0.00000	0.00000
	high-DNAJB1	0.00228	0.00010		high-DNAJB1	0.00000	0.00000
K137-K507	ATP-state, no dilution	0.00155	0.00006	K557-K601	ATP-state, no dilution	0.00175	0.00018
	ADP-state, no dilution	0.00453	0.00096		ADP-state, no dilution	0.00185	0.00040
	low-DNAJB1	0.00223	0.00017		low-DNAJB1	0.00000	0.00000
	high-DNAJB1	0.00395	0.00044		high-DNAJB1	0.00000	0.00000
K458-K507	ATP-state, no dilution	0.02330	0.00334	K88-K102	ATP-state, no dilution	0.00483	0.00064
	ADP-state, no dilution	0.03368	0.00253		ADP-state, no dilution	0.00365	0.00085
	low-DNAJB1	0.02188	0.00065		low-DNAJB1	0.00000	0.00000
	high-DNAJB1	0.03841	0.00152		high-DNAJB1	0.00000	0.00000
K88-K246	ATP-state, no dilution	0.03889	0.00081	K88-K126	ATP-state, no dilution	0.00269	0.00035
	ADP-state, no dilution	0.01081	0.00235		ADP-state, no dilution	0.00184	0.00036
	low-DNAJB1	0.00358	0.00056		low-DNAJB1	0.00000	0.00000
	high-DNAJB1	0.00446	0.00002		high-DNAJB1	0.00000	0.00000
K451-K535	ATP-state, no dilution	0.00605	0.00067	K526-K573	ATP-state, no dilution	0.00572	0.00001
	ADP-state, no dilution	0.01090	0.00193		ADP-state, no dilution	0.00747	0.00121
	low-DNAJB1	0.00916	0.00005		low-DNAJB1	0.00000	0.00000
	high-DNAJB1	0.01395	0.00022		high-DNAJB1	0.00000	0.00000
K526-K561	ATP-state, no dilution	0.00109	0.00015	K569-K601	ATP-state, no dilution	0.00531	0.00014
	ADP-state, no dilution	0.00136	0.00025		ADP-state, no dilution	0.00698	0.00173
	low-DNAJB1	0.00131	0.00011		low-DNAJB1	0.00000	0.00000
	high-DNAJB1	0.00135	0.00034		high-DNAJB1	0.00000	0.00000

XL	Experiment	XL relative occurrence	XL relative occurrence SD	XL	Experiment	XL relative occurrence	XL relative occurrence SD
Nterm-K512	ATP-state, no dilution	0.00000	0.00000	K535-K550	ATP-state, no dilution	0.00611	0.00023
	ADP-state, no dilution	0.00660	0.00084		ADP-state, no dilution	0.00286	0.00071
	low-DNAJB1	0.00686	0.00097		low-DNAJB1	0.00000	0.00000
	high-DNAJB1	0.00867	0.00023		high-DNAJB1	0.00000	0.00000
K137-K512	ATP-state, no dilution	0.00141	0.00014	K137-K159	ATP-state, no dilution	0.00313	0.00033
	ADP-state, no dilution	0.00596	0.00104		ADP-state, no dilution	0.00248	0.00040
	low-DNAJB1	0.00229	0.00052		low-DNAJB1	0.00000	0.00000
	high-DNAJB1	0.00521	0.00073		high-DNAJB1	0.00000	0.00000
K112-K524	ATP-state, no dilution	0.01286	0.00080	K345-K361	ATP-state, no dilution	0.00455	0.00016
	ADP-state, no dilution	0.00912	0.00175		ADP-state, no dilution	0.00233	0.00031
	low-DNAJB1	0.00237	0.00050		low-DNAJB1	0.00000	0.00000
	high-DNAJB1	0.00207	0.00020		high-DNAJB1	0.00000	0.00000
K569-K609	ATP-state, no dilution	0.00996	0.00073	K539-K550	ATP-state, no dilution	0.01391	0.00072
	ADP-state, no dilution	0.01084	0.00057		ADP-state, no dilution	0.00431	0.00146
	low-DNAJB1	0.00043	0.00005		low-DNAJB1	0.00000	0.00000
	high-DNAJB1	0.00000	0.00000		high-DNAJB1	0.00000	0.00000
K500-K512	ATP-state, no dilution	0.02071	0.00097	K550-K567	ATP-state, no dilution	0.00854	0.00106
	ADP-state, no dilution	0.00949	0.00136		ADP-state, no dilution	0.01527	0.01247
	low-DNAJB1	0.00146	0.00016		low-DNAJB1	0.00000	0.00000
	high-DNAJB1	0.00000	0.00000		high-DNAJB1	0.00000	0.00000
K328-K345	ATP-state, no dilution	0.00433	0.00093	K187-K357	ATP-state, no dilution	0.00385	0.00016
	ADP-state, no dilution	0.00176	0.00009		ADP-state, no dilution	0.00184	0.00032
	low-DNAJB1	0.00082	0.00018		low-DNAJB1	0.00000	0.00000
	high-DNAJB1	0.00000	0.00000		high-DNAJB1	0.00000	0.00000

XL	Experiment	XL relative occurrence	XL relative occurrence SD	XL	Experiment	XL relative occurrence	XL relative occurrence SD
K71-K88	ATP-state, no dilution	0.00731	0.00011	K159-K188	ATP-state, no dilution	0.00000	0.00000
	ADP-state, no dilution	0.00387	0.00077		ADP-state, no dilution	0.00216	0.00077
	low-DNAJB1	0.00116	0.00004		low-DNAJB1	0.00082	0.00016
	high-DNAJB1	0.00000	0.00000		high-DNAJB1	0.00000	0.00000
K112-K159	ATP-state, no dilution	0.00384	0.00039	Ntertm-K507	ATP-state, no dilution	0.00000	0.00000
	ADP-state, no dilution	0.00475	0.00047		ADP-state, no dilution	0.00279	0.00055
	low-DNAJB1	0.00192	0.00021		low-DNAJB1	0.00666	0.00078
	high-DNAJB1	0.00000	0.00000		high-DNAJB1	0.00791	0.00008
K126-K137	ATP-state, no dilution	0.00836	0.00100	K112-K507	ATP-state, no dilution	0.00000	0.00000
	ADP-state, no dilution	0.00605	0.00052		ADP-state, no dilution	0.00742	0.00138
	low-DNAJB1	0.00070	0.00004		low-DNAJB1	0.00000	0.00000
	high-DNAJB1	0.00000	0.00000		high-DNAJB1	0.00215	0.00019
K573-K589	ATP-state, no dilution	0.00218	0.00020	K246-K257	ATP-state, no dilution	0.00000	0.00000
	ADP-state, no dilution	0.00000	0.00000		ADP-state, no dilution	0.00000	0.00000
	low-DNAJB1	0.00254	0.00036		low-DNAJB1	0.00218	0.00032
	high-DNAJB1	0.00273	0.00028		high-DNAJB1	0.00290	0.00010
K557-K567	ATP-state, no dilution	0.00382	0.00025	K188-K507	ATP-state, no dilution	0.00000	0.00000
	ADP-state, no dilution	0.00000	0.00000		ADP-state, no dilution	0.00000	0.00000
	low-DNAJB1	0.00644	0.00054		low-DNAJB1	0.00092	0.00002
	high-DNAJB1	0.00711	0.00021		high-DNAJB1	0.00118	0.00014
K56-K257	ATP-state, no dilution	0.00312	0.00011	K137-K524	ATP-state, no dilution	0.00000	0.00000
	ADP-state, no dilution	0.00000	0.00000		ADP-state, no dilution	0.00000	0.00000
	low-DNAJB1	0.00106	0.00013		low-DNAJB1	0.00101	0.00010
	high-DNAJB1	0.00116	0.00006		high-DNAJB1	0.00179	0.00025
K159-K187	ATP-state, no dilution	0.00000	0.00000	K137-K526	ATP-state, no dilution	0.00000	0.00000
	ADP-state, no dilution	0.00427	0.00114		ADP-state, no dilution	0.00000	0.00000
	low-DNAJB1	0.00058	0.00008		low-DNAJB1	0.00098	0.00013
	high-DNAJB1	0.00082	0.00010		high-DNAJB1	0.00174	0.00021

XL	Experiment	XL relative occurrence	XL relative occurrence SD	XL	Experiment	XL relative occurrence	XL relative occurrence SD
K108-K271	ATP-state, no dilution	0.00000	0.00000	K415-K526	ATP-state, no dilution	0.00000	0.00000
	ADP-state, no dilution	0.00165	0.00021		ADP-state, no dilution	0.00000	0.00000
	low-DNAJB1	0.00192	0.00010		low-DNAJB1	0.00096	0.00016
	high-DNAJB1	0.00225	0.00037		high-DNAJB1	0.00189	0.00039
K250-K500	ATP-state, no dilution	0.00000	0.00000	K583-K597	ATP-state, no dilution	0.00000	0.00000
	ADP-state, no dilution	0.00085	0.00018		ADP-state, no dilution	0.00000	0.00000
	low-DNAJB1	0.00146	0.00010		low-DNAJB1	0.00464	0.00056
	high-DNAJB1	0.00179	0.00003		high-DNAJB1	0.00572	0.00040
K187-K512	ATP-state, no dilution	0.00000	0.00000	Nterm-K187	ATP-state, no dilution	0.00000	0.00000
	ADP-state, no dilution	0.00382	0.00033		ADP-state, no dilution	0.00000	0.00000
	low-DNAJB1	0.00319	0.00021		low-DNAJB1	0.00477	0.00069
	high-DNAJB1	0.00488	0.00004		high-DNAJB1	0.00623	0.00043

Unique Inter-XL											
ATP-state, no dilution			ATP-state, with dilution			low-DNAJB1			high-DNAJB1		
XL	Inter/intra ratio	Inter/intra ratio SD	XL	Inter/intra ratio	Inter/intra ratio SD	XL	Inter/intra ratio	Inter/intra ratio SD	XL	Inter/intra ratio	Inter/intra ratio SD
K159-K271	0.2156	0.0019	K56-K271	0.231	0.026	K112-K345	0.951	0.036	K108-K345	0.854	0.035
K451-K512	0.1220	0.0031	K187-K319	0.243	0.009	K188-K458	0.637	0.039	K246-K597	0.859	0.047
K71-K250	0.2833	0.0176	K137-K497	0.341	0.035	Nterm-K507	0.007	0.001	K88-K524	0.757	0.081
K451-K507	0.1575	0.0123	K415-K512	0.229	0.005	K137-K526	0.277	0.009			
K159-K188	0.1021	0.0127				K497-K557	0.592	0.062			
K159-K325	0.2552	0.0074				K159-K458	0.528	0.011			
K159-K257	0.1888	0.0857				K159-K524	0.151	0.004			
K187-K345	0.2881	0.0330									
K71-K257	0.2212	0.0220									
K71-K507	0.3278	0.0337									
K159-K319	0.2629	0.0162									
K56-K325	0.3373	0.0083									
K71-K451	0.3210	0.0159									
K159-K187	0.1353	0.0176									
K71-K159	0.2555	0.0253									
K126-K187	0.3178	0.0113									
K271-K423	0.1960	0.0168									
K56-K187	0.2634	0.0052									
K137-K319	0.2632	0.0244									
K56-K328	0.3272	0.0220									

Ambiguous Inter-XL					
**XL**	**Experiment**	**XL relative occurrence**	**XL relative occurrence SD**	**Inter/intra ratio**	**Inter/intra ratio SD**
K345-K557	ATP-state, no dilution	0.00118	0.00013	0.935	0.023
	ADP-state, no dilution	0.00625	0.00206	0.935	0.030
	low-DNAJB1	0.00191	0.00029	0.868	0.024
	high-DNAJB1	0.00126	0.00008	0.740	0.035
K159-K345	ATP-state, no dilution	0.00305	0.00015	0.312	0.015
	ADP-state, no dilution	0.00368	0.00084	0.554	0.038
	low-DNAJB1	0.01014	0.00005	0.943	0.020
	high-DNAJB1	0.00833	0.00008	0.963	0.004
K137-K187	ATP-state, no dilution	0.00675	0.00007	0.644	0.006
	ADP-state, no dilution	0.00659	0.00077	0.782	0.029
	low-DNAJB1	0.00055	0.00004	0.915	0.063
	high-DNAJB1	0.00044	0.00010	0.891	0.045
K137-K188	ATP-state, no dilution	0.00399	0.00001	0.553	0.011
	ADP-state, no dilution	0.00660	0.00243	0.764	0.051
	low-DNAJB1	0.00106	0.00012	0.739	0.034
	high-DNAJB1	0.00000	0.00000	0.000	0.000
K328-K557	ATP-state, no dilution	0.00174	0.00016	0.987	0.021
	ADP-state, no dilution	0.00233	0.00090	0.963	0.022
	low-DNAJB1	0.00063	0.00005	0.890	0.049
	high-DNAJB1	0.00000	0.00000	0.000	0.000
K137-K458	ATP-state, no dilution	0.00000	0.00000	0.000	0.000
	ADP-state, no dilution	0.00185	0.00024	0.408	0.027
	low-DNAJB1	0.00090	0.00011	0.607	0.028
	high-DNAJB1	0.00285	0.00021	0.592	0.008

XL	Experiment	XL relative occurrence	XL relative occurrence SD	Inter/intra ratio	Inter/intra ratio SD
K159-K451	ATP-state, no dilution	0.00000	0.00000	0.000	0.000
	ADP-state, no dilution	0.00138	0.00029	0.283	0.012
	low-DNAJB1	0.00097	0.00003	0.449	0.019
	high-DNAJB1	0.00096	0.00010	0.440	0.047
K250-K583	ATP-state, no dilution	0.00083	0.00009	0.909	0.046
	ADP-state, no dilution	0.00090	0.00023	0.855	0.164
	low-DNAJB1	0.00000	0.00000	0.000	0.000
	high-DNAJB1	0.00000	0.00000	0.000	0.000
K71-K319	ATP-state, no dilution	0.00272	0.00008	0.285	0.012
	ADP-state, no dilution	0.00130	0.00025	0.314	0.037
	low-DNAJB1	0.00000	0.00000	0.000	0.000
	high-DNAJB1	0.00000	0.00000	0.000	0.000
K187-K271	ATP-state, no dilution	0.00310	0.00015	0.190	0.011
	ADP-state, no dilution	0.00183	0.00044	0.215	0.032
	low-DNAJB1	0.00000	0.00000	0.000	0.000
	high-DNAJB1	0.00000	0.00000	0.000	0.000
K137-K271	ATP-state, no dilution	0.00253	0.00013	0.224	0.016
	ADP-state, no dilution	0.00168	0.00029	0.269	0.046
	low-DNAJB1	0.00000	0.00000	0.000	0.000
	high-DNAJB1	0.00000	0.00000	0.000	0.000
Nterm-K187	ATP-state, no dilution	0.00321	0.00007	0.219	0.012
	ADP-state, no dilution	0.00710	0.00200	0.151	0.029
	low-DNAJB1	0.00000	0.00000	0.000	0.000
	high-DNAJB1	0.00000	0.00000	0.000	0.000

XL	Experiment	XL relative occurrence	XL relative occurrence SD	Inter/intra ratio	Inter/intra ratio SD
K500-K561	ATP-state, no dilution	0.00127	0.00007	0.089	0.023
	ADP-state, no dilution	0.00212	0.00033	0.267	0.033
	low-DNAJB1	0.00000	0.00000	0.000	0.000
	high-DNAJB1	0.00000	0.00000	0.000	0.000
K126-K361	ATP-state, no dilution	0.00101	0.00023	0.815	0.040
	ADP-state, no dilution	0.00146	0.00023	0.924	0.037
	low-DNAJB1	0.00000	0.00000	0.000	0.000
	high-DNAJB1	0.00000	0.00000	0.000	0.000
K71-K271	ATP-state, no dilution	0.00354	0.00010	0.255	0.011
	ADP-state, no dilution	0.00174	0.00067	0.290	0.019
	low-DNAJB1	0.00000	0.00000	0.000	0.000
	high-DNAJB1	0.00000	0.00000	0.000	0.000
Nterm-K497	ATP-state, no dilution	0.00000	0.00000	0.000	0.000
	ADP-state, no dilution	0.00000	0.00000	0.000	0.000
	low-DNAJB1	0.00108	0.00010	0.485	0.038
	high-DNAJB1	0.00091	0.00014	0.631	0.221
K159-K497	ATP-state, no dilution	0.00000	0.00000	0.000	0.000
	ADP-state, no dilution	0.00000	0.00000	0.000	0.000
	low-DNAJB1	0.00190	0.00006	0.389	0.018
	high-DNAJB1	0.00179	0.00012	0.417	0.007
K187-K497	ATP-state, no dilution	0.00000	0.00000	0.000	0.000
	ADP-state, no dilution	0.00000	0.00000	0.000	0.000
	low-DNAJB1	0.00119	0.00010	0.586	0.041
	high-DNAJB1	0.00136	0.00006	0.540	0.030

XL	Experiment	XL relative occurrence	XL relative occurrence SD	Inter/intra ratio	Inter/intra ratio SD
Nterm-K345	ATP-state, no dilution	0.00000	0.00000	0.000	0.000
	ADP-state, no dilution	0.00000	0.00000	0.000	0.000
	low-DNAJB1	0.00131	0.00010	0.871	0.026
	high-DNAJB1	0.00137	0.00001	0.916	0.029
K345-K524	ATP-state, no dilution	0.00000	0.00000	0.000	0.000
	ADP-state, no dilution	0.00000	0.00000	0.000	0.000
	low-DNAJB1	0.00181	0.00006	0.701	0.057
	high-DNAJB1	0.00196	0.00023	0.758	0.056
K187-K458	ATP-state, no dilution	0.00000	0.00000	0.000	0.000
	ADP-state, no dilution	0.00000	0.00000	0.000	0.000
	low-DNAJB1	0.00071	0.00004	0.539	0.033
	high-DNAJB1	0.00100	0.00009	0.559	0.010
K345-K597	ATP-state, no dilution	0.00000	0.00000	0.000	0.000
	ADP-state, no dilution	0.00000	0.00000	0.000	0.000
	low-DNAJB1	0.00052	0.00005	0.828	0.054
	high-DNAJB1	0.00059	0.00004	0.852	0.055
Nterm-K458	ATP-state, no dilution	0.00000	0.00000	0.000	0.000
	ADP-state, no dilution	0.00000	0.00000	0.000	0.000
	low-DNAJB1	0.00218	0.00010	0.432	0.041
	high-DNAJB1	0.00246	0.00002	0.402	0.011
K137-K524	ATP-state, no dilution	0.00000	0.00000	0.000	0.000
	ADP-state, no dilution	0.00000	0.00000	0.000	0.000
	low-DNAJB1	0.00097	0.00006	0.225	0.011
	high-DNAJB1	0.00145	0.00013	0.255	0.027

XL	Experiment	XL relative occurrence	XL relative occurrence SD	Inter/intra ratio	Inter/intra ratio SD
K88-K345	ATP-state, no dilution	0.00000	0.00000	0.000	0.000
	ADP-state, no dilution	0.00000	0.00000	0.000	0.000
	low-DNAJB1	0.00054	0.00005	0.925	0.006
	high-DNAJB1	0.00059	0.00002	1.006	0.037
K345-K458	ATP-state, no dilution	0.00000	0.00000	0.000	0.000
	ADP-state, no dilution	0.00000	0.00000	0.000	0.000
	low-DNAJB1	0.00125	0.00012	0.640	0.031
	high-DNAJB1	0.00130	0.00015	0.640	0.007

Complex modelling*		
**low-DNAJB1**	**high-DNAJB1**	**Common**
K500-K37	K345-K286	Nterm-K286
K257-Nterm	K497-K286	K345-K35
	K512-K286	K108-K286
	K524-K286	K187-K286
	K458-K21	K458-K35
	K159-K35	K112-K286
	K458-K286	
	K88-K286	

*First residue - HSC70, second residue - DNAJB1

Table S3: The ANOVA statistics for the HSc70 Intra-Xl relative abundances frotm Table S2 (Hsc70 Intra-ls, Ambiguous XL)

XL	F_value	p_value	FDR
K88-K246	340.265	8.97E-09	5.11E-07
K569-K609	252.793	1.61E-06	3.23E-05
K56-K257	248.267	1.70E-06	3.23E-05
K500-K512	199.722	3.24E-06	4.62E-05
K112-K524	56.277	1.01E-05	1.15E-04
K71-K88	91.326	3.22E-05	3.06E-04
K56-K71	33.399	7.13E-05	4.52E-04
K126-K137	69.699	7.03E-05	4.52E-04
K187-K328	300.964	6.48E-05	4.52E-04
Nterm-K137	28.126	1.34E-04	7.61E-04
K458-K507	25.391	1.93E-04	9.17E-04
Nterm-K507	50.021	1.81E-04	9.17E-04
K557-K567	46.914	2.17E-04	9.52E-04
K451-K535	20.883	3.86E-04	1.55E-03
K137-K512	20.554	4.08E-04	1.55E-03
K112-K159	32.246	6.17E-04	2.20E-03
K112-K126	29.197	8.09E-04	2.71E-03
K345-K357	16.545	8.61E-04	2.73E-03
K345-K361	75.446	9.67E-04	2.90E-03
K187-K507	25.859	1.12E-03	3.20E-03
K539-K550	67.688	1.19E-03	3.23E-03
K187-K512	24.354	1.32E-03	3.42E-03
K250-K500	23.231	1.50E-03	3.71E-03
K451-K531	13.541	1.68E-03	3.82E-03
K137-K507	13.494	1.70E-03	3.82E-03
K187-K357	55.385	1.74E-03	3.82E-03
K328-K345	21.505	1.83E-03	3.87E-03
K159-K187	19.684	2.31E-03	4.71E-03
K159-K507	11.406	2.92E-03	5.74E-03
K535-K550	37.012	3.69E-03	7.01E-03
K112-K507	28.598	5.89E-03	1.08E-02
Nterm-K159	8.199	8.00E-03	1.43E-02
K188-K357	10.816	1.02E-02	1.77E-02
K88-K257	7.186	1.17E-02	1.96E-02
K137-K526	17.286	1.42E-02	2.31E-02
K137-K524	15.559	1.69E-02	2.68E-02
K415-K526	12.014	2.57E-02	3.96E-02
K108-K126	11.449	2.77E-02	4.15E-02
K108-K524	6.770	2.89E-02	4.23E-02
K246-K257	9.383	3.75E-02	5.35E-02
K188-K507	9.143	3.90E-02	5.42E-02
XL	F_value	p_value	FDR

Nterm-K187	8.065	4.69E-02	6.36E-02
K328-K357	4.042	5.07E-02	6.72E-02
Nterm-K512	4.454	6.52E-02	8.44E-02
K159-K188	6.255	6.67E-02	8.45E-02
K3-K187	5.921	7.17E-02	8.78E-02
K88-K126	5.878	7.24E-02	8.78E-02
K526-K573	4.263	1.08E-01	1.28E-01
K108-K271	3.128	1.17E-01	1.36E-01
K583-K597	3.896	1.20E-01	1.36E-01
K137-K159	2.455	1.92E-01	2.15E-01
K88-K102	2.198	2.12E-01	2.33E-01
K569-K601	2.065	2.24E-01	2.41E-01
K573-K589	1.411	3.15E-01	3.32E-01
K550-K567	0.666	4.60E-01	4.77E-01
K526-K561	0.568	6.51E-01	6.63E-01
K557-K601	0.108	7.58E-01	7.58E-01
